# Reprogramming to Pluripotency Using Designer TALE Transcription Factors Targeting Enhancers

**DOI:** 10.1016/j.stemcr.2013.06.002

**Published:** 2013-07-11

**Authors:** Xuefei Gao, Jian Yang, Jason C.H. Tsang, Jolene Ooi, Donghai Wu, Pentao Liu

**Affiliations:** 1 Wellcome Trust Sanger Institute, Hinxton, Cambridge CB10 1HH, UK; 2 Key Laboratory of Regenerative Biology, Guangzhou Institute of Biomedicine and Health, Chinese Academy of Sciences, Guangzhou 510530, China

## Abstract

The modular DNA recognition code of the transcription-activator-like effectors (TALEs) from plant pathogenic bacterial genus *Xanthomonas* provides a powerful genetic tool to create designer transcription factors (dTFs) targeting specific DNA sequences for manipulating gene expression. Previous studies have suggested critical roles of enhancers in gene regulation and reprogramming. Here, we report dTF activator targeting the distal enhancer of the *Pou5f1* (*Oct4*) locus induces epigenetic changes, reactivates its expression, and substitutes exogenous OCT4 in reprogramming mouse embryonic fibroblast cells (MEFs) to induced pluripotent stem cells (iPSCs). Similarly, dTF activator targeting a *Nanog* enhancer activates *Nanog* expression and reprograms epiblast stem cells (EpiSCs) to iPSCs. Conversely, dTF repressors targeting the same genetic elements inhibit expression of these loci, and effectively block reprogramming. This study indicates that dTFs targeting specific enhancers can be used to study other biological processes such as transdifferentiation or directed differentiation of stem cells.

## Highlights

• The dTF for the *Oct4* distal enhancer replaces OCT4 in reprogramming MEFs to iPSCs
• The repressor dTF induces ESC differentiation and blocks reprogramming
• The dTF for the *Nanog* reprograms EpiSCs to iPSCs, whereas the repressor blocks it
• Targeting enhancers by dTFs can be an effective way to control gene expression

## Graphical Abstract

**Figure:**
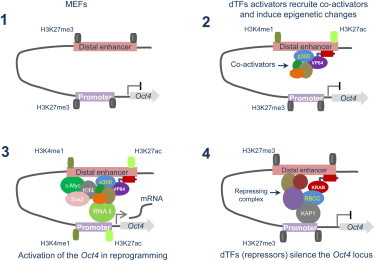

## Introduction

Proper gene expression is a central part of development and a key to cellular homeostasis. Transcription factors (TFs) control gene expression, and a subset of them are regarded as master regulators for lineage development and/or identity maintenance (Spitz and Furlong, 2012). Master regulators often modulate gene expression through enhancers, which are important genetic elements that control the spatial and temporal expression of specific sets of genes (Levine, 2010). Epigenetic patterning of enhancers by the intricate interplay between DNA methylation, specific TFs binding, and histone modifications has been demonstrated to occur before cell-fate decisions (Spitz and Furlong, 2012). Therefore, we hypothesized that a more effective and physiologically relevant regulation of gene expression can be achieved by direct manipulation of specific enhancers.

Transcriptional-activator-like effectors (TALEs) are natural effector proteins secreted by plant pathogenic bacteria to modulate gene expression in host plants and to facilitate bacterial infection. TALEs contain a modular DNA binding domain consisting of highly similar tandem repeats of 33–35 amino acids. The specificity of nucleotide recognition of each repeat is determined by two hypervariable amino acids at positions 12 and 13 (Boch et al., 2009; Cong et al., 2012; Moscou and Bogdanove, 2009; Streubel et al., 2012). The simple coding rule makes TALEs a unique tool to generate programmable effectors targeting a genomic region (Bogdanove and Voytas, 2011). TALE-based designer transcription activators (A-dTF) or repressors (R-dTF) have been constructed by linking TALEs to activation or repression domains, respectively. These dTFs target specific promoters based on the assumption that the close proximity of the dTFs to the transcription start site (TSS) would modulate transcription (Bultmann et al., 2012; Geissler et al., 2011; Morbitzer et al., 2010; Zhang et al., 2011). Attempts were made to use A-dTFs to activate endogenous pluripotency loci such as *Sox2*, *Klf4*, *Oct4*, and *c-Myc* (Bartsevich et al., 2003; Bultmann et al., 2012; Juárez-Moreno et al., 2013; Zhang et al., 2011). For the *Oct4* locus, these experiments achieved modest activation but failed to demonstrate any physiological impact in reprogramming or other cellular processes.

In this study, we chose the *Oct4* and *Nanog* loci to investigate whether dTFs could regulate gene expression via their specific enhancers and whether the activation or repression could impact reprogramming to induced pluripotent stem cells (iPSCs) or affect embryonic stem (ES) cell differentiation. We report here that direct regulation of the endogenous pluripotency loci by dTFs targeting enhancers enables reprogramming of mouse embryonic fibroblast cells (MEFs)or epiblast stem cells (EpiSCs) to iPSCs in the absence of exogenous reprogramming factors OCT4 or NANOG. Therefore, dTFs targeting enhancers of genomic loci encoding key regulators can provide an effective approach for reprogramming to pluripotency and potentially for other applications such as transdifferentiation and directed differentiation of stem cells.

## Results

### A-dTFs Targeting the *Oct4* Distal Enhancer Activate the Locus

**Figure 1:**
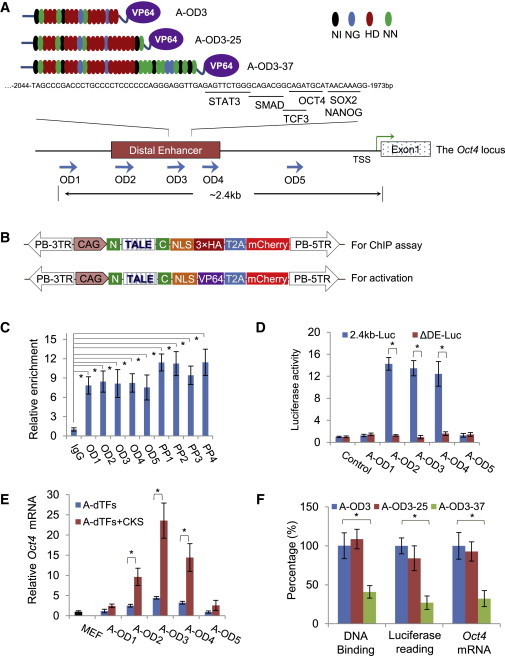
Reactivation of the *Oct4* Locus by dTFs (A) The schematic diagram of TALE proteins and their binding sites at the *Oct4* distal enhancer (DE). Color code for the amino acid positions 12 and 13 in a TALE repeat and the corresponding nucleotide in DNA: black NI for A, blue NG for T, red HD for C, and green NN for G. TALE proteins OD2, OD3, and OD4 bind inside the DE region, whereas OD1 and OD5 bind outside the DE. (B) Cloning of TALE protein coding DNA or dTFs into the PB vector. For ChIP analysis testing binding of TALEs to their target sequences, 3 × HA tag was added at the C terminus of TALE proteins (upper panel). For activator dTFs (A-dTFs), the VP64 was added (lower panel). In all cases, mCherry was coexpressed with TALE proteins or dTFs via the T2A. N and C are the N and C termini of the TALE protein. CAG: the CAG promoter. PB-5TR and PB-3TR are the two ends of the PB transposon. NLS, nuclear localization signal. (C) Validation of TALE binding to the *Oct4* locus in ChIP assay using an antibody to HA tag followed by qPCR amplifying the corresponding genomic DNAs. IgG was used as the control. (D) Luciferase assays to measure dTF activities. The 2.4 kb-Luc reporter has the DE, PE, and PP of the *Oct4* locus, whereas the ΔDE construct lacks the DE. (E) qRT-PCR analysis of *Oct4* mRNA levels in MEFs expressing the activator dTFs (A-dTFs) alone or plus CKS. All gene expression values are normalized to the expression of *Gapdh*. (F) Comparison of three dTFs, OD3, OD3-25, and OD3-37, on DNA binding, luciferase activities, and *Oct4* mRNA levels induced by them in MEFs. Results are representative of three independent experiments and are mean ± SD, n = 3. ^∗^p < 0.01. See also Figure S1 and Table S1.

We chose the mouse *Oct4* locus to test functionality of dTFs because it is an essential pluripotency factor (Nichols et al., 1998), and reactivation of the locus is a critical step in reprogramming of somatic cells to iPSCs (Polo et al., 2012; Takahashi and Yamanaka, 2006). Specific genetic elements are shown to regulate proper *Oct4* expression in stem cells of distinct pluripotent states (Bao et al., 2009; Minucci et al., 1996; Okazawa et al., 1991; Yeom et al., 1996), namely, the distal enhancer (DE) in murine ES cells and germ cells, the proximal enhancer (PE) in EpiSCs, and the proximal promoter (PP). Because the DE is active specifically in ES cells, we constructed five dTFs to target five 19 bp sequences (OD1–OD5) inside or outside the DE (Figure 1A; Table S1 available online). OD3 is on the 5′ side of the multiple transcription factor binding sites of STAT3, TCF3, OCT4, SOX2, and NANOG in the DE (Chen et al., 2008; Young, 2011) (Figure 1A). We also targeted four 19 bp sequences (PP1–PP4) in the *Oct4* promoter as controls (Figure S1A). The TALE DNA binding domains were constructed using a modified Golden Gate cloning system (Sanjana et al., 2012) (Figure S1B). We next evaluated the DNA-binding property of these TALE proteins. To provide quantitation of binding, we fused 3 × hemagglutinin (HA) tags with the C terminus of each TALEs (Figure 1B, upper panel). Chromatin immunoprecipitation (ChIP) quantitative real-time PCR analysis of ES cells after expression of the HA-tagged TALEs for 2 days showed all nine TALE proteins bound their intended sequences as indicated by the 7- to 12-fold enrichment compared to the immunoglobulin (Ig) G control (Figure 1C).

We next investigated the ability of A-dTFs to activate the *Oct4* locus. To make A-dTFs, we fused VP64 (Beerli et al., 1998) to the C terminus of the TALE proteins in a *piggyBac* delivery vector (Wang et al., 2008). The fusion protein is linked to mCherry by T2A peptide for convenient tracking of TALE protein expression (Figure 1B, lower panel). We first examined the ability of A-dTFs to activate *Oct4* expression in luciferase assay. MEFs were cotransfected with vectors expressing dTFs and a luciferase construct containing the 2.4 kb region covering all three upstream regulatory elements of the *Oct4* locus (Figure S1A). Two days after transfection, three A-dTFs targeting the DE (A-OD2, A-OD3, and A-OD4) and three A-dTFs targeting the promoter (A-PP1–A-PP3) substantially enhanced luciferase activities compared to the control construct (Figures 1D and S1C). Once the DE was deleted in the luciferase reporter (ΔDE in Figure S1A), none of the A-dTFs targeting the DE was able to activate the luciferase reporter indicating specificity of A-dTFs for the DE (Figure 1D). On the contrary, A-PP1, A-PP2, and A-PP3 still activated the reporter carrying only the *Oct4* promoter region (Figure S1C). Consistent with the luciferase assay, expression of A-OD2, A-OD3, and A-OD4 in MEFs for 48 hr increased the *Oct4* mRNA by 3- to 4-fold measured by quantitative RT-PCR (qRT-PCR) (Figure 1E), whereas all four dTFs targeting the promoter achieved lower mRNA levels, in contrast to the luciferase assay (Figure S1D). The *Oct4* locus is silenced in MEFs through repressor complexes, chromatin modifications, and DNA methylation. Three of the four transcription factors for reprogramming somatic cells to iPSCs, C-MYC, KLF4, and SOX2, are suggested to have roles in chromatin remodeling or bind the DE of the *Oct4* locus (Yamanaka, 2008; Young, 2011). Coexpressing A-OD2/3/4 with C-MYC, KLF4, and SOX2 (CKS) in MEFs for 48 hr caused 10- to 20-fold increase of *Oct4* mRNA with A-OD3 being the most potent (Figure 1E), indicating a synergistic interaction of dTFs with these transcription factors. In contrast, coexpressing A-PP1–A-PP4 with CKS failed to substantially increase *Oct4* mRNA levels (Figure S1D), highlighting the significance of targeting the enhancer, rather than the promoter, in regulating gene expression by dTFs. In an attempt to further improve the potency of A-OD3, we made two new dTFs (A-OD3-25 and A-OD-37) recognizing 25 and 37 bp sequences encompassing the sequence bound by A-OD3 (Figure 1A and Table S1). A-OD3-25 showed the similar DNA binding ability and promoted *Oct4* mRNA expression and higher luciferase activities as compared to A-OD3, whereas A-OD3-37 was not competent in both assays (Figure 1F), suggesting that excessive peptide repeats may cause unnatural protein structure because naturally found TALEs have around 20 peptide repeats (Boch et al., 2009; Moscou and Bogdanove, 2009). To exclude the possibility that expression of the endogenous *Oct4* in MEFs was due to general chromatin remodeling in the *Oct4* genomic region by the VP64 domain, we examined expression of several neighboring genomic loci on the mouse chromosome 17, including *Tcf19*, *Cchcr1*, and *H2Q-10* (Figure S1E), as well as *Kcnk18*, which has a stretch of DNA sequence (ACCCTGCCCCTCC) that is similar to the 19 bp region targeted by A-OD3 as shown in Figure 1A. The expression of these four loci were not substantially altered either by expression of A-OD3 alone or in combination with CKS (Figure S1F).

### Activation of the *Oct4* Locus by dTFs Reprograms MEFs to iPSCs in Concert with C-MYC, KLF4, and SOX2

**Figure 2:**
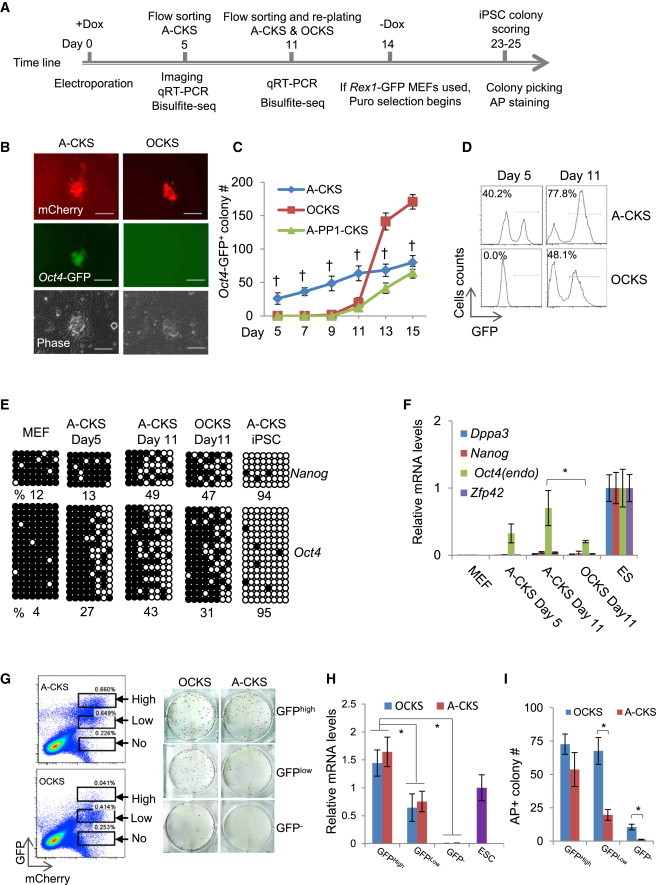
A-OD3 Replaces Exogenous OCT4 in Reprogramming *Oct4*-GFP MEFs to iPSCs (A) The time line for reprogramming MEFs to iPSCs using dTFs. MEFs under reprogramming were analyzed at several time points for various assay. The iPSC colonies were scored and picked on day 23 or 25. (B) Activation of the endogenous *Oct4* locus detected by GFP expression. mCherry^+^ cells were imaged on day 5 after transfection. Scale bar: 200 μm. (C) Quantitation of GFP^+^ colonies from MEFs expressing dTFs targeting the DE (A-OD3) or the promoter (A-PP1) at various time points of reprogramming. (D) mCherry^+^ cells were analyzed for GFP expression in flow cytometry on days 5 and 11. (E and F) The GFP^+^ cells were harvested by flow sorting and analyzed for DNA demethylation in the *Oct4* and *Nanog* promoters and for gene expression. The percentages in (E) are the demethylated CpG in the promoters. (G) The reprogramming potential of MEFs with a reactivated *Oct4* locus. *Oct4*-GFP MEFs under reprogramming were sorted into three populations based on GFP intensity on day 11. Six hundreds cells of each population were replated into a 6-well plate to allow colony formation. (H) Endogenous *Oct4* expression in the three cell populations measured by qRT-PCR. (I) AP^+^ colony numbers from the replated cells scored on day 25. All gene expression levels are normalized to *Gapdh*. Results are representatives of three independent experiments and are mean ± SD. n = 3. ^∗^p < 0.01. ^†^p < 0.05 A-CKS compared to OCKS. See also Figure S2 and Tables S2, S3, and S4.

We next explored whether the endogenous *Oct4* activation induced by dTFs has functional consequences and attempted reprogramming MEFs to iPSCs in the absence of exogenous OCT4. The PB (*piggyBac*) vectors containing doxcycline (Dox)-inducible dTFs and CKS were delivered to Oct4-GFP reporter MEFs via the *piggyBac* transposition (Figure S2A) (Wang et al., 2011). Expression of the reprogramming factors was induced by adding Dox in the medium (Figure 2A). PB transposition is efficient in mammalian cells (Wang et al., 2011); approximately 4% of MEFs survived electroporation and expressed the transgenes in the genome. As early as 5 days after transfection and Dox induction, microscopic GFP^+^ colonies (also mCherry^+^) were visually identifiable in the combination of A-OD3 plus CKS or A-CKS (Figure 2B), whereas no GFP^+^ colonies were found in the control combination of exogenous OCT4 plus CKS (or OCKS) or A-PPs plus CKS, until day 11. We thus chose A-OD3 in the subsequent characterization of its function in reprogramming and in ES cells.

Despite the rapid reactivation of endogenous *Oct4* expression, A-OD3 was not sufficient to substantially enhance the reprogramming process compared to exogenous OCT4 because the combination OCKS caught up in terms of GFP^+^ colonies number at the late stage of reprogramming. On day 13, there were on average 68 GFP^+^ colonies in A-CKS dish compared to 141 colonies in the OCKS combination in three independent experiments (Figure 2C), consistent with the notion that high exogenous OCT4 levels facilitate late stages of reprogramming (Carey et al., 2011). On the other hand, although none of A-PPs in combination with CKS caused rapid *Oct4* locus reactivation, they eventually produced GFP^+^ colonies, with a reprogramming pattern similar to OCKS but with fewer colonies (Figure 2C; Table S2). The result suggested that A-PPs were also capable of inducing endogenous *Oct4* reactivation in cooperation with CKS despite of a slower kinetics, potentially due to lower OCT4 expression. On day 5 and 11, 40% and 78% mCherry^+^ cells expressing A-CKS became GFP^+^. Expression of the endogenous OCT4 was confirmed in mCherry^+^ MEFs by immunostaining (Figure S2B). In contrast, no GFP^+^ cells were detected in cells expressing OCKS on day 5 and only 48% mCherry^+^ cells became GFP^+^ on day 11 (Figure 2D).

To further investigate reactivation of the *Oct4* locus by A-OD3, the GFP^+^ cells from *Oct4*-GFP MEFs were harvested by fluorescence-activated cell sorting (FACS) and analyzed as soon as they appeared. In the day 5 GFP^+^ cells reprogrammed by A-CKS, the *Oct4* promoter started to be demethylated, but the locus was the only one activated among several pluripotency loci examined include *Nanog*, *Zfp42* (*Rex1*), and *Dppa3* (*Stella*) (Figures 2E and 2F). On the other hand, on day 11, GFP^+^ cells of both A-CKS and OCKS expressed low levels of key pluripotent genes besides the endogenous *Oct4* (Figure 2F). Moreover, DNA demethylation was detected in the promoters of both the *Oct4* and *Nanog* (Figure 2E). Therefore, rapid reactivation of the *Oct4* locus facilitated by A-OD3 represents a necessary yet insufficient step in reprogramming. Additional epigenetic barriers at other key pluripotency loci still need to be overcome at the late stage of reprogramming (Plath and Lowry, 2011).

Nevertheless, reactivation of the endogenous *Oct4* locus by A-OD3 in MEFs under reprogramming marked the cells that were destined to become iPSCs. We flow-sorted cells expressing either A-CKS or OCKS (mCherry^+^) into three cell populations, GFP^high^, GFP^low^, and GFP^−^, on day 11. Cells were collected, counted, and replated (600 cells) on feeder cells to allow them to continue reprogramming (Figures 2A and 2G). qRT-PCR analysis confirmed the correlation between GFP expression and the endogenous Oct4 mRNA level (Figure 2H). Interestingly, in cells expressing A-CKS, the GFP^high^ cells formed 53 AP^+^ colonies (70% of the total colonies), and the rest (about 20 AP^+^ colonies) originated from GFP^low^ cells (Figure 2I). The GFP^−^ cells did not produce any colonies. On the other hand, AP^+^ colonies were formed from all the three cell populations expressing OCKS, with 48% (72) from GFP^high^, 45% (67) from GFP^low^, and 7% (10) from GFP^−^ cells (Figure 2I). These results demonstrated that the levels of the endogenous *Oct4* expression induced by the dTF were more predictive for successful reprogramming compared to expressing exogenous *Oct4*.

Endogenous *Oct4* activation is a critical and major limiting step in somatic cell reprogramming (Boiani et al., 2002; Hochedlinger and Plath, 2009). To investigate whether the reactivation of the endogenous *Oct4* locus by A-OD3 could enhance reprogramming of somatic cells by the standard four Yamanaka factors OCKS, we cotransfected *Oct4*-GFP reporter MEFs with Dox-inducible expression vectors of OCKS and A-OD3 (A-OCKS). Coexpression of these factors produced GFP^+^ cells as early as 3 days after Dox induction (Figure S2C), indicating an even faster reactivation of the *Oct4* locus comparing to A-CKS. Additionally, A-OCKS also produced more AP^+^ colonies (Figure S2D).

**Figure 3:**
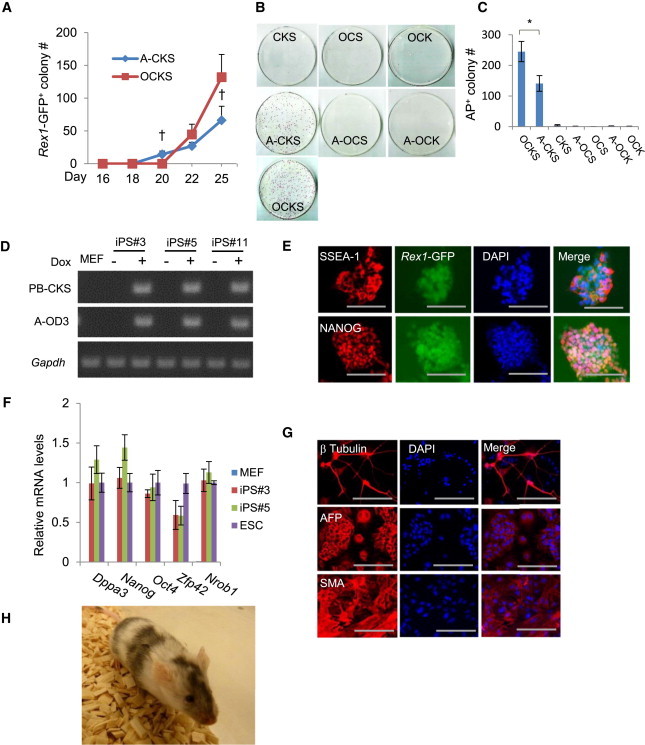
Characterization of iPSCs Reprogrammed by A-CKS (A) GFP^+^ colonies from *Rex1*-GFP MEFs by A-CKS or OCKS at several time points during reprogramming. (B and C) Reprogramming of *Rex1*-GFP MEFs using various combinations of A-OD3 and the Yamanaka factors. Dox-independent Puro^+^ colonies were scored 25 days after transfection. (D) Detection of leaky expression in iPSC lines reprogrammed using Dox-inducible A-CKS. Primers specific for the exogenous CKS or for A-OD3 were used in RT-PCR. The three lines shown have no detectable exogenous factor expression in the absence of Dox. (E) Immunostaining of iPSC colonies for NANOG and SSEA1. DNA was stained with propidium iodide. Scale bars: 200.0 μm. (F) qRT-PCR analysis of expression of several pluripotency genes in iPSC line #3 and #5 reprogrammed by A-CKS. (G) iPSCs reprogrammed by A-CKS are able to differentiate to cells of all three germ layers in vitro. Antibodies used are as follows: neuron-specific class III β-tubulin; SMA (smooth muscle α-actin) and AFP (α-fetoprotein). Scale bars: 200.0 μm. (H) Chimera mouse generated using iPSC line #3 expression of *Gapdh* was used as the control in RT-PCR. Results are representatives of three independent experiments and are mean ± SD. n = 3. ^∗^p < 0.01. ^†^p < 0.05 A-CKS compared to OCKS. See also Figure S3 and Tables S3 and S4.

*Rex1* is expressed in mouse ES cells but not in EpiSCs and represents a better marker for ground-state pluripotency or for monitoring late stages of reprogramming (Brons et al., 2007; Tesar et al., 2007; Toyooka et al., 2008). To further demonstrate A-OD3’s function in reprogramming, we repeated the experiments using the *Rex1*-GFP reporter MEFs where the *GFP-IRES-Puro* cassette was inserted into the *Rex1* locus (Guo et al., 2011). iPSCs from these MEFs would be both GFP^+^ and Puro^r^. In contrast to the rapid reactivation of the *Oct4* locus in the aforementioned experiments, A-CKS only slightly accelerated reactivation of the *Rex1* locus in the reporter MEFs, with GFP^+^ colonies appearing on day 20 compared to day 22 for the OCKS control (Figure 3A), again demonstrating that rapid reactivation of the *Oct4* locus alone by A-OD3 is an early event in reprogramming. Dox was subsequently withdrawn after 14 days to select for Dox- or exogenous-factor-independent colonies (Figure 2A). A-CKS produced around 60 Puro^r^ or GFP^+^ colonies per transfection of one million MEFs, whereas OCKS transfection produced about 120 such colonies (Figure 3A). On the other hand, A-OD3 was unable to effectively substitute either SOX2 or KLF4 in reprogramming (Figures 3B and 3C).

We next examined *Rex1* locus reactivation by A-OD3 plus OCKS or A-OCKS. Again *Rex1*-GFP^+^ colony appeared on day 20, slightly earlier than the OCKS control (Figures S3A and S3B). Reprogramming *Rex1*-GFP MEFs by A-OCKS also consistently led to roughly 1.5-fold more Puro^+^ colonies than expressing OCKS alone (Figure S3A). Therefore, even in the presence of exogenous *Oct4*, early reactivation of the endogenous *Oct4* locus by the dTF still promoted reprogramming.

### iPSC Reprogrammed by A-CKS Are Pluripotent

From the Puro^r^ iPSCs colonies produced by A-CKS, we picked 36 for characterization. From these 36 lines, seven were found not to express any of the exogenous reprogramming factors (Figure 3D), whereas the other lines still had expression due to leakiness of the Tet/On system. These nonleaky iPSCs were characterized in vitro and in vivo for their pluripotency. Both immunostaining and qRT-PCR analyses demonstrated that these exogenous-factor- independent iPSC lines expressed key pluripotency genes at levels comparable to that in mouse ES cells (Figures 3E and 3F). The iPSCs retained the normal karyotype after 16 passages (Figure S3C). In vitro differentiation of the iPSCs produced somatic cell types representing all three germ layers (Figure 3G). Finally, chimeric mice were obtained using these iPSCs confirming their pluripotency in normal development (Figure 3H).

### The dTF Activator A-OD3 Causes Rapid Histone Modification Changes at the *Oct4* Locus

In ES cells, the *Oct4* locus is marked by active histone modifications such as H3K27 acetylation and H3K4 trimethylation, whereas, in MEFs, the *Oct4* locus is transcriptionally repressed and is tightly packaged into nucleosomes marked by H3K9me3 and H3K27me3 (Mikkelsen et al., 2007).

**Figure 4:**
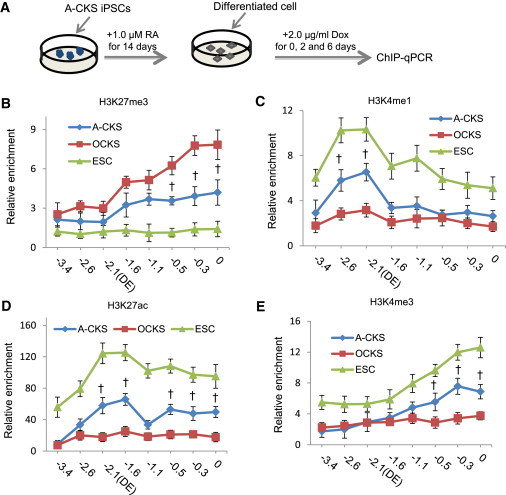
Changes of Histone H3 Modifications at the *Oct4* Locus Induced by A-OD3 (A) Differentiation of iPSCs produced by Dox-inducible OCKS or A-CKS and reexpression of the exogenous factors. (B–E) Histone H3 modifications H3K27me3 (B), H3K4me1 (C), H3K27ac (D), and H3K4me3 (E), were analyzed in the ChIP assay followed by qPCR. The relative enrichments were normalized to IgG, and a genomic region at the *Tyr* locus was used as the unrelated locus control. Values in x axis indicate the locations of PCR primers used qPCR in the ChIP assay. −0.3: 0.3 kb upstream of the TSS. Results are representative of three independent experiments in three cell lines and are mean ± SD. n = 3. †p < 0.05 A-CKS compared to OCKS. See also Figure S4 and Table S3.

We next investigated the impacts of A-OD3 on histone modifications at the *Oct4* locus. To this end, we differentiated iPSC lines obtained by using Dox-inducible A-CKS or OCKS by all *trans*-retinoic acid for 14 days, and all the differentiated cells lost expression of pluripotency markers. Expression of the reprogramming factors was then induced, and cells were collected on days 0, 2, and 6 for ChIP analysis (Figure 4A).

Many putative enhancer elements have been mapped in the genomes by their association with specific histone modifications (Ong and Corces, 2011). We examined H3K4me1, H3K4me3, H3K27me3, and H3K27ac at eight specific sites in the 3.4 kb region upstream of the *Oct4* locus TSS by ChIP assay. This genomic region encompasses the DE, PE, and PP. Compared to the OCKS, expression of A-CKS rapidly reduced H3K27me3 levels (Figure 4B) concomitant with increased levels of the active markers H3K4me1 (Figure 4C), H3K27ac (Figure 4D), and H3K4me3 (Figure 4E), as early as 2 days after Dox induction. In contrast, OCKS only induced similar changes six days after Dox induction (Figures S4A–S4D).

### The dTF Repressor R-OD3 Targeting the *Oct4* Distal Enhancer Induces ES Cell Differentiation

The effectiveness of A-OD3 to reactivate the *Oct4* locus prompted us to investigate whether a repressor targeting the same genetic element could negatively regulate the locus. We replaced the VP64 domain in A-OD3 and A-OD1 with the KRAB repressor domain of KOX1 (Margolin et al., 1994) to make mCherry-tagged Dox-inducible R-OD3 and R-OD1, which targets a region upstream of the distal enhancer as a control.

**Figure 5:**
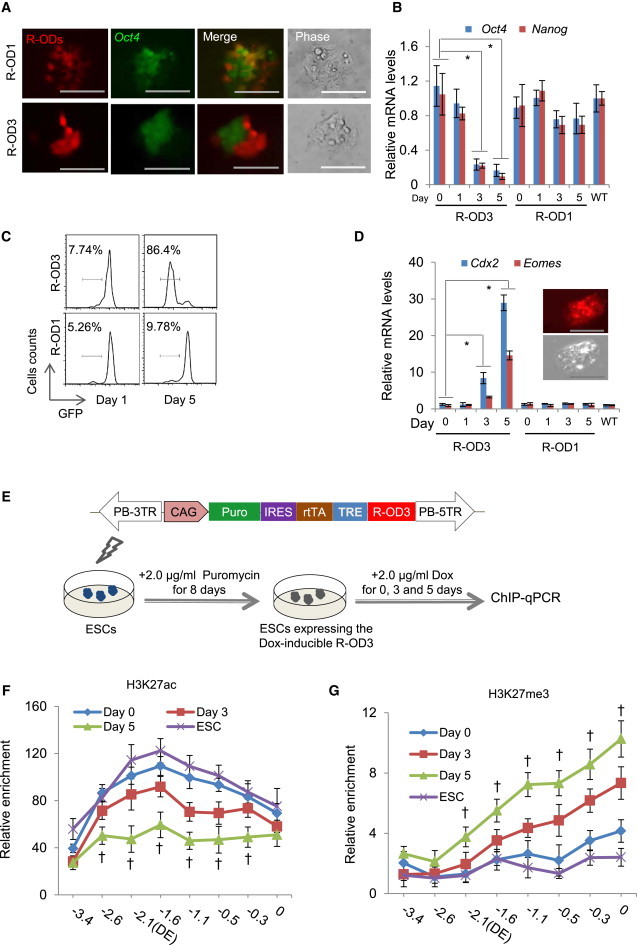
Repressor dTF R-OD3 Blocks the *Oct4* Locus Expression (A) Images of *Oct4*-GFP ES cells expressing two repressor dTFs: R-OD3 and R-OD1. Cells expressing dTFs are mCherry^+^. (B) *Oct4* and *Nanog* expression in ES cells expressing R-OD3 or R-OD1 detected in qRT-PCR. (C) Flow cytometric analysis of Oct4-GFP ES cells on days 1 and 5 following expression of repressor dTFs (gated for mCherry^+^). (D) Differentiation of ES cells to trophoblast-like cells caused by R-OD3 and expression of *Cdx2* and *Eomes* in these cells. (E) Diagram showing the PB vector expressing Dox-inducible R-OD3 for making a stable ES cell line. The repressor R-OD1 serves as the negative control. (F and G) Epigenetic changes at the *Oct4* locus in ES cells expressing R-OD3 for 3 days measured in ChIP assay at the *Oct4* locus. The relative enrichments were normalized to IgG, and a genomic region at the *Tyr* locus was used as the unrelated locus control. Values in x axis indicate the locations of PCR primers used in ChIP assay. −0.3: 0.3 kb upstream of the TSS. Scale bars: 200 μm. Results are representative of three independent lines and are mean ± SD. n = 3. ^∗^p < 0.01. ^†^p < 0.05 day 5 compared to day 0. See also Figure S5 and Tables S3 and S4.

We next tested the repressors in *Oct4*-GFP ES cells. In cells expressing R-OD3, the mCherry^+^ cells became GFP^dim^ or GFP^−^ as soon as 3 days after Dox induction (Figure 5A). In contrast, R-OD1 had no obvious effect because the mCherry^+^ ES cells were still GFP^+^.

We harvested mCherry^+^ cells by FACS at different time points of Dox induction and analyzed expression of *Oct4* via either GFP expression or transcription level. After 3 days of R-OD3 expression, *Oct4* mRNA levels were substantially decreased, and, on day 5, it was at about 10% of that in wild-type ES cells (Figure 5B). Flow cytometric analysis confirmed that on day 5, 86% of mCherry^+^ ES cells became GFP^−^ (Figure 5C). Concomitantly, *Nanog*, which is a target of OCT4, was also markedly downregulated in ES cells expressing R-OD3 (Figure 5B). By contrast, expression of R-OD1 did not noticeably decrease *Oct4* mRNA or substantially increase GFP^−^ cells (Figures 5B and 5C). Morphologically, the mCherry^+^GFP^−^ cells differentiated into trophectoderm-like cells and expressed high levels of *Cdx2* and *Eomes* (Nichols et al., 1998; Niwa et al., 2005) (Figure 5D). ChIP analysis showed that ES cells stably expressing R-OD3 for 3 days (Figure 5E) had decreased levels of H3K27ac and increased H3K27me3 at the *Oct4* locus, indicating silencing of the locus (Figures 5F and 5G). Expression of R-OD1, on the other hand, did not cause similar changes (Figures S5A and S5B). These results clearly demonstrated the effectiveness of the dTF repressor and also confirmed the essential role of the *Oct4* distal enhancer in pluripotency.

### The dTF Repressor R-OD3 Targeting the *Oct4* Distal Enhancer Blocks Reprogramming

**Figure 6:**
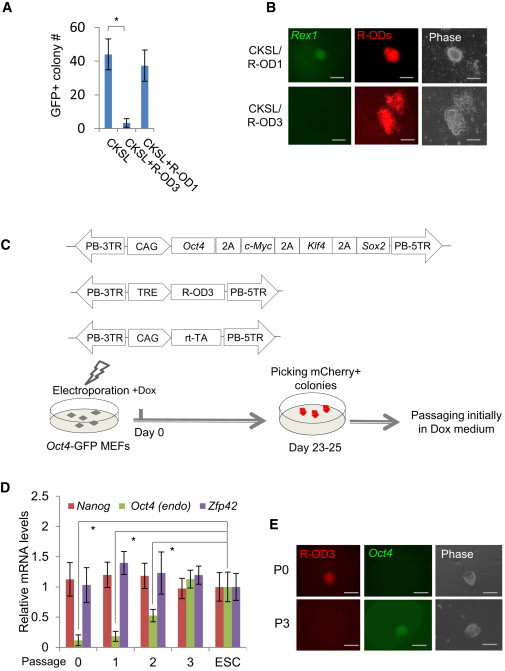
R-OD3 Suppresses the *Oct4* Locus and Blocks Reprogramming (A) Reprogramming of *Rex1*-GFP MEFs to iPSCs by CKS plus LRH1 (CKSL) in the presence of repressor dTF R-OD3 or R-OD1. (B) The small number of colonies reprogrammed by CKSL in the presence of R-OD3 (mCherry^+^) were all GFP^−^, indicating blocking of reprogramming. (C) Reprogramming of *Oct4*-GFP MEFs using CAG-OCKS and Dox-inducible R-ODs. mCherry^+^ iPSC colonies were picked and expanded in the presence of Dox. (D) Analysis of expression of endogenous *Oct4*, *Nanog*, and *Zfp42* (*Rex1*) in iPSCs reprogrammed in (C) in either the presence (passage 0) or absence of Dox (passages 1–3). Expression in ES cells was used as the control. (E) Reactivation of the *Oct4* locus monitored by GFP expression in iPSCs obtained in (C) once Dox was withdrawn. iPSCs became mCherry^−^ and GFP^+^ within three passages. All gene expression values are normalized to the expression of *Gapdh*. Scale bars: 200 μm. Results are representative of three independent experiments and are mean ± SD. n = 3. ^∗^p < 0.01. See also Figure S6 and Table S4.

The effective repression of the *Oct4* locus by R-OD3 provided an opportunity to examine the consequence of keeping the *Oct4* locus inactive in reprogramming. Two experimental approaches were taken. In the first case, we reprogrammed *Rex1*-GFP MEFs by expressing CKS and LRH1 (CKSL) under the constitutive active CAG promoter as LRH1 is reported to replace exogenous OCT4 in reprogramming by binding and activating the *Oct4* locus (Heng et al., 2010). Expressing CKSL produced 44 GFP^+^ colonies scored 22 days after induction (Figure 6A), whereas coexpression of R-OD3 with CKSL produced no mCherry^+^GFP^+^ colonies (Figure 6B). Suppression of the *Oct4* distal enhancer by R-OD3 therefore effectively blocked reprogramming. R-OD1, on the other hand, did not affect reprogramming.

In the second approach, we reprogrammed *Oct4*-GFP MEFs by CAG-OCKS (constitutive expression) and Dox-inducible R-OD3 (Figure 6C). In the presence of exogenous OCT4, reprogramming was not affected by R-OD3 (Figure S6). The iPSCs obtained expressed pluripotency genes at levels comparable to that in ES cells (Passage 0 in Figure 6D), except endogenous *Oct4*, which was suppressed by R-OD3. It further confirmed the effectiveness of repression of the *Oct4* locus by R-OD3.

We next examined the reversibility of R-OD3 repression on the *Oct4* locus by withdrawing Dox and thus turning off R-OD3 expression in iPSCs obtained above. The endogenous *Oct4* mRNA gradually reached 30% of that in ES cells at passage 2 and reached similar levels at passage 3 (Figure 6D) as the cells switched from mCherry^+^/GFP^−^ to mCherry^−^/GFP^+^ (Figure 6E). However, it should be noted that the continuous expression of exogenous factors in these iPSCs could influence the repression reversibility in this experiment.

### Regulation of the *Nanog* Locus by dTFs Targeting the 5 kb Enhancer

We next extended our findings of enhancer regulation by dTFs to the *Nanog* locus. Studies of *Nanog* expression regulation have revealed an enhancer located at approximately 5.0 kb upstream of its TSS, which is a DNase I-hypersensitive site and bound by OCT4, NANOG, SOX2, and ZFP281 (Levasseur et al., 2008; Loh et al., 2006). We first made three A-dTFs (A-ND1, A-ND2, and A–ND3) targeting the respective 19 bp sequences inside or outside the 5 kb enhancer region (Figure S7A; Table S1). Luciferase assay in MEFs showed that A-ND2 could increase luciferase activities by more than 3-fold compared to the control (Figure S7B).

**Figure 7:**
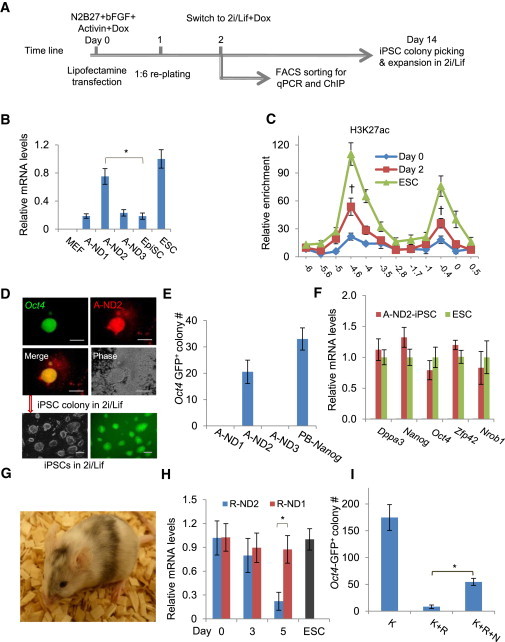
Regulation of the *Nanog* Locus by dTFs Targeting the 5 kb Enhancer (A) Expression of dTFs in reprogramming EpiSCs. The transfected EpiSCs were collected on day 2 for several assays including qPCR, qRT-PCR, and ChIP analysis, or allowed to be reprogrammed to iPSCs. (B) *Nanog* mRNA levels in EpiSCs expressing A-NDs. (C) H3K27ac levels at the *Nanog* locus in EpiSCs expressing A-NDs. (D) Reprogramming *Oct4*-GFP reporter EpiSCs to iPSCs by A-ND2. (E) GFP^+^ iPSC colonies from EpiSCs by A-ND2. (F) qRT-PCR analysis of several pluripotency genes in iPSCs reprogrammed by A-ND2. (G) Chimera derived from iPSCs from EpiSCs by A-ND2. (H) Decrease of *Nanog* mRNA levels in ES cells expressing R-ND2 in qRT-PCR analysis. (I) Efficient reprogramming of EpiSCs to iPSCs by *Klf4* (K), which was suppressed by R-ND2 (R). Expressing a *Nanog* transgene rescues reprogramming (K+N+R-ND2). All gene expression values are normalized to the expression of the Gapdh gene. Scale bars: 200.0 μm. Results are representative of three independent experiments and are mean ± SD. n = 3. ^∗^p < 0.01. ^†^p < 0.05 day 2 compared to day 0. See also Figure S7 and Tables S3 and S4.

EpiSCs are pluripotent cells established from developing epiblasts and express lower levels of NANOG (Guo et al., 2009; Silva et al., 2009). Exogenous *Nanog* transgene reprograms EpiSCs to naive iPSCs (Silva et al., 2009). To examine whether A-NDs were able to increase *Nanog* expression in EpiSCs and perhaps also to reprogram EpiSCs to iPSC, we transfected EpiSCs by lipofection with a PB construct expressing the Dox-inducible A-NDs (Figure 7A). The transfection and the PB transposition efficiencies were estimated to be about 15% and 1%–2%, respectively. Among the three dTFs, A-ND2 increased *Nanog* expression by 3-fold and reached comparable *Nanog* levels found in mouse ES cells (Figure 7B). A-ND2 also caused rapid epigenetic changes at the *Nanog* locus with H3K27ac levels being substantially increased only 2 days induction (Figure 7C), whereas A-ND1 had no obvious effects (Figure S7C). We then investigated A-ND2 in reprogramming *Oct4*-GFP EpiSCs (Guo et al., 2009) to iPSCs (Figure 7A). Expression of A-ND2 produced 21 iPSC colonies compared to 30 colonies when exogenous *Nanog* was expressed (Figures 7D and 7E). A-ND1 or A-ND3, however, did not yield any colony. iPSC lines generated by A-ND2 expressed key pluripotency genes at levels comparable to that in mouse ES cells (Figure 7F). Adult chimeras were also derived from these iPSCs (Figure 7G). Therefore, the dTF targeting to the *Nanog* 5 kb enhancer was able to activate the locus and reprogram EpiSCs to iPSCs.

We also made R-ND2 from A-ND2 by replacing the VP64 domain with the KRAB domain and investigated *Nanog* expression in ES cells. We transfected Nanog-GFP reporter mouse ES cells cultured in serum containing medium with the mCherry-tagged R-ND2 PB transgene. In flow cytometric analysis, more than 80% of mCherry^+^ ES cells became GFP^−/dim^ in 5 days indicating loss of *Nanog* expression (Figure S7D). Suppression of *Nanog* expression was confirmed in qRT-PCR, which showed that only 25% of *Nanog* mRNA left in cells expressing A-ND2 for 5 days (Figure 7H). The essential functions of NANOG for acquisition of ground-state or naive pluripotency have been demonstrated in *Nanog*-deficient ES cells (Silva et al., 2006, 2009). EpiSCs express little KLF4. Exogenous KLF4 reprograms EpiSCs to naive iPSCs (Guo et al., 2009). We re-examined the requirement of NANOG in KLF4-mediated EpiSCs reprogramming to iPSCs using R-ND2. We introduced a PB-CAG-*Klf4* transgene to EpiSCs via the PB transposition, which produced around 170 iPSC colonies scored on day 14 (Figure 7I). By contrast, if R-ND2 was coexpressed with the *Klf4* transgene (*Klf4* plus R-ND2 or K+R), fewer than ten colonies were obtained (Figure 7I), and none of them were mCherry^+^GFP^+^. Repressing *Nanog* by R-ND2 in KLF4-mediated EpiSC reprogramming was partially rescued using a *Nanog* transgene (K+R+N) (Figure 7I), confirming the essential function of NANOG in reprogramming EpiSCs to iPSCs.

In summary, targeting the *Nanog* 5 kb enhancer by dTFs also enabled manipulation of the endogenous locus for reprogramming to pluripotency.

## Discussion

Regulation of gene expression is central in development and in homeostasis and is achieved by both *cis*- and *trans*-elements. Enhancers dictate the spatial and temporal patterns of gene expression during development and can drive progenitor cells to distinct cell fates. Recent studies have shown that cell-fate decisions and lineage commitment are regulated by epigenetic patterning at enhancers (Ong and Corces, 2011). One of the best-characterized enhancers in ES cells is the distal enhancer of the *Oct4* locus, which controls *Oct4* expression in ES cells and PGCs (Bao et al., 2009; Minucci et al., 1996; Yeom et al., 1996) and is marked by active histone modifications and bound by multiple key pluripotency transcription factors (Chen et al., 2008; Young, 2011). We decided to target the *Oct4* distal enhancer as a proof of principle for dTFs to regulate a key pluripotency locus. Previous attempts to activate the *Oct4* expression were focused on targeting dTFs to the promoter, which only activated its expression in reporter assays but not effectively in MEFs or other somatic cells (Bultmann et al., 2012; Zhang et al., 2011), an observation that we were able to reproduce in this study. In contrast, A-OD3, which targets the region close to the binding sites of OCT4, SOX2, and NANOG at the distal enhancer, induces rapid histone modification changes and efficiently reactivates the locus in MEFs. These results are consistent with a recent study that, in reprogramming, OCT4, SOX2, and KLF4 act as pioneer factors at enhancers of genes that promote reprogramming (Soufi et al., 2012). Indeed, A-OD3, working together with SOX2, KLF4, and C-MYC, reprograms MEFs to bona fide iPSCs, bypassing the need of exogenous OCT4. Furthermore, the rapid reactivation of endogenous OCT4 by A-OD3 enhances reprogramming in the context of exogenous OCT4, SOX2, KLF4, and C-MYC. Besides replacing exogenous OCT4, using A-OD3 has helped reveal new insight in reprogramming. Endogenous *Oct4* reactivation is believed to be an essential landmark and a bottleneck step for reacquisition of pluripotency (Kim et al., 2009) and being the only reprogramming factor recalcitrant to substitution by a family member (Nakagawa et al., 2008). Yet, we show here that early reactivation of the *Oct4* locus alone by dTFs is not sufficient to complete reprogramming. Additional epigenetic changes in other pluripotency loci are still required despite robust reactivation of endogenous *Oct4* in MEF cells.

We used the VP64 transactivation domain to generate dTF activators. VP64 at the *Oct4* distal enhancer would presumably recruit and interact with histone acetyltransferase p300 and transcriptional activation complexes (Ito et al., 2000; Milbradt et al., 2011) and induce epigenetic changes that facilitate binding of additional factors such as OCT4 itself at the distal enhancer. Replacing VP64 with the KRAB domain in the dTFs produces repressor dTFs. R-OD3 suppresses the *Oct4* locus and induces ES cell differentiation and blocks reprogramming. The repression by R-OD3 could be reversed by coexpressing exogenous OCT4, SOX2, KLF4, and C-MYC in iPSCs. In addition to the *Oct4* locus, a dTF targeting to the 5 kb *Nanog* enhancer also allows efficient regulation of this locus. The activator alone reprograms EpiSCs to iPSCs, whereas the repressor suppresses *Nanog* expression and permits dissection of NANOG requirements in reprogramming.

This proof-of-principle study demonstrates that targeting key regulatory elements such as enhancers of key genes is an effective way to regulate their expression. dTFs could mimic the complicated transcription regulation by recruiting physiologically relevant factors to a specific locus. Reprogramming somatic cells to iPSCs is an inefficient process. Using dTFs rather than native transcription factors could eventually prove to be an alternative or even more efficient reprogramming approach. It can be envisioned that in the future a combination of dTFs (activators and repressors) targeting loci encoding master regulators could enable cellular transdifferentiation or direct stem cells to a specific cell lineage as master regulators for a number of lineages have been extensively studied. Two recent studies reported that one could achieve tunable gene activation by combinations of dTFs targeting the promoters (Maeder et al., 2013; Perez-Pinera et al., 2013); a similar approach may also be feasible to regulate enhancers.

With the advances of next-generation sequencing technologies, genome-wide mapping of regulatory elements have identified thousands of enhancers and other elements (Shen et al., 2012). Functional validation of these enhancers to investigate their roles in specific cell types or developmental stages presents a challenge. Advances in TALE cloning technologies now enable high-throughput assembly of dTFs at low cost (Reyon et al., 2012). dTFs may therefore provide a solution to functionally dissect the newly identified enhancers, including the recently reported “super-enhancers” (Whyte et al., 2013), in vitro or in vivo.

## Experimental Procedures

### Mice

Housing and breeding of mice and experimental procedures using mice were according to the UK 1986 Animals Scientific Procedure Act and local institute ethics committee regulations.

### Mouse ES and iPSC Culture

Mouse ES cells and iPSCs were normally cultured in M15 medium: knockout DMEM, 15% FBS (HyClone), 1 × glutamine-penicillin-streptomycin (Invitrogen), 1 × Nonessential Amino Acids (NEAA; Invitrogen), 0.1 mM β-mercaptoethanol (β-ME; Sigma), and 10^6^ U/ml LIF (Millipore). We also cultured iPSCs in the chemically defined medium N2B27/2i/LIF.

### Transfection of MEFs and Reprogramming to iPSCs

MEFs were prepared from 13.5 day postcoitum mouse embryos and were cultured in M10 (knockout DMEM plus 10% of fetal calf serum). MEFs were transfected by Amaxa Nucleofector (Lonza) program A-023 and were seeded on STO feeder cells for reprogramming.

### EpiSC Culture and Reprogramming

Established *Oct4*-GFP reporter EpiSCs (Guo et al., 2009) were cultured in N2B27/Activin/bFGF at the density of 6 × 10^5^ cells per well in a 6-well plate coated with human fibronectin for Lipofectamine 2000 (Invitrogen) transfection. Twenty-four hours after transfection, EpiSCs were split at 1:6 in 6-well plate and cultured in EpiSC culture medium containing Dox (2 μg/ml) for 1 day before the culture medium was changed to N2B27/2i/LIF and Dox (2 μg/ml). The medium was changed every 2 days. The GFP^+^ iPSC colonies were counted on day 14 posttransfection. Transfection and PB transposition efficiencies were calculated similar to in MEFs.

### ChIP Analysis

IgG and antibodies for the HA tag, H3K4me3, H3K4me1, H3K27ac, and H3K27me3 were used for ChIP analysis.

### Statistical Analysis

Statistical significance was determined using a Student’s t test with two-tailed distribution. p values < 0.05 were considered as significant. Data are shown as mean **±** SD.

Supplemental Information and Tables S1–S5 include further details of materials and methods.

## Supplemental Information

Document S1. Supplemental Experimental Procedures, Figures S1–S7, and Tables S1–S5
