# Supplementary material for: Reprogramming to Pluripotency Using Designer TALE Transcription Factors Targeting Enhancers
Source: Stem Cell Reports. 2013 Jul 11;1(2):183–97. doi: 10.1016/j.stemcr.2013.06.002 (PMC3757749; doi:10.1016/j.stemcr.2013.06.002)
Supplement: Document S1. Supplemental Experimental Procedures, Figures S1–S7, and Tables S1–S5 [file mmc1.pdf]

Stem Cell Reports, Volume *1*

Supplemental Information

Reprogramming to Pluripotency Using Designer TALE Transcription Factors Targeting Enhancers

Xuefei Gao, Jian Yang, Jason Tsang, Jolene Ooi, Donghai Wu, and Pentao Liu

Supplementary Figures

Figure S1

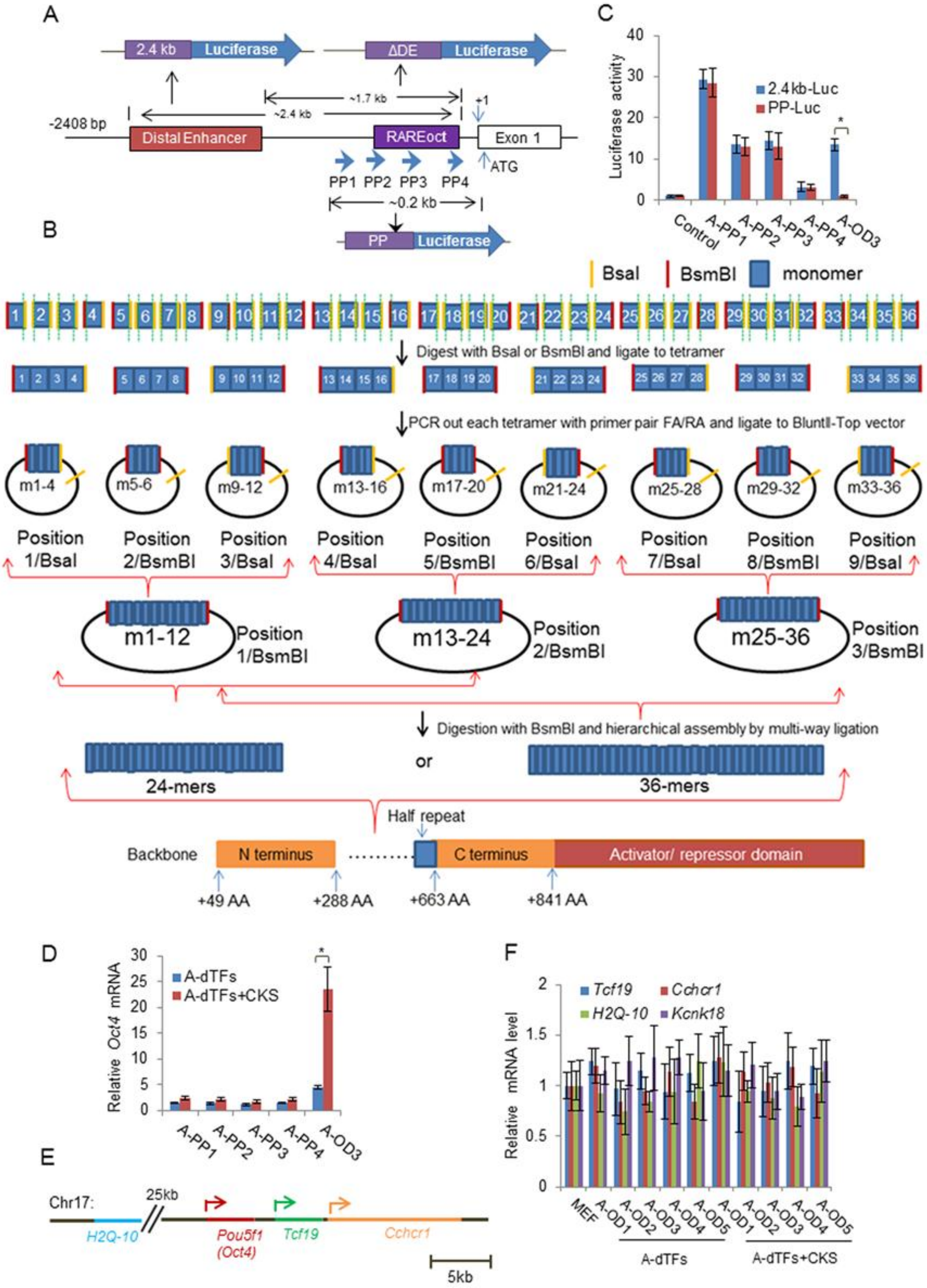

Figure S1. Assembly and functional assessment of dTFs. Related to Figure 1. (A) The diagram showing luciferase assay reporter constructs carrying the 2.4kb genomic DNA (DE, PE and PP), the 1.7kb genomic DNA (PE and PP) or ΔDE, and 0.2kb promoter. The binding sites for the 4 TALE proteins PP1-4 targeting the *Oct4* promoter were also showed. RAREoct, RA-responsive element. (B) For making DNA binding domains with up to 36 TALE repeats, thirty-six separate PCRs were performed for each of the four types of repeat monomers (NI, HD, NG and NN) to generate a set of 36 monomers which served as the assembly starting materials (Table S3 and Table S5). Each of the 36 PCR products for a given monomer type had a unique and compatible linker specifying its programmable position in sequential ligation. For #24 monomer PCR, F12/R13 and F12/R14 primer pairs were used respectively for 24 and 36 repeats assembling (Table S5). After enzymatic digestion with a type II restriction endonuclease (BsaI and BsmBI), 4 monomers were ligated to make a tetramer. A tetramer was amplified by PCR with primer pair FA/RA (Table S3) and ligates into Blunt-Top vector for sequencing confirmation and long-term storage. Three tetramers were joined together by a three-way ligation to make 12-mers in Blunt II-Top vector. To construct 24 or 36 repeats, two or three 12-mers were cut out from the vectors and cloned into a backbone plasmid containing the N and C termini of the TALE protein. The resultant constructs had DNA binding domains for 25 bp or 37 bp (plus the last half repeat) DNA. The N-terminus (aa49-288) and C-terminus truncation (aa663-841) of TALE Hax3 protein were used as the TALE architecture. (C) Luciferase activities in MEFs co-transfected by A-PPs and PP-Luc reporter construct were quantitated 48 hours after transfection. A-OD3 was used as a control. (D) Activation of the *Oct4* locus assessed by *Oct4* mRNA expression in MEFs using either A-dTFs alone or in combination with CKS. A-OD3 targeting the DE was much more potent than any of the A-dTFs binding the promoter (A-PP1/2/3/4). (E) The schematic diagram describing the distance between *Oct4* and the three surrounding loci: *Tcf19*, *Cchcr1*, and *H2Q-10*. (F) Expression of four loci in MEFs expressing dTFs targeting the *Oct4* locus. The *Kcnk18* locus has a stretch of DNA sequence (ACCCTGCCCCTCC) that is similar to the 19 bp region targeted by A-OD3. Results are representative of three independent experiments and are means ± S.D., n=3. **p* <0.01.

Figure S2

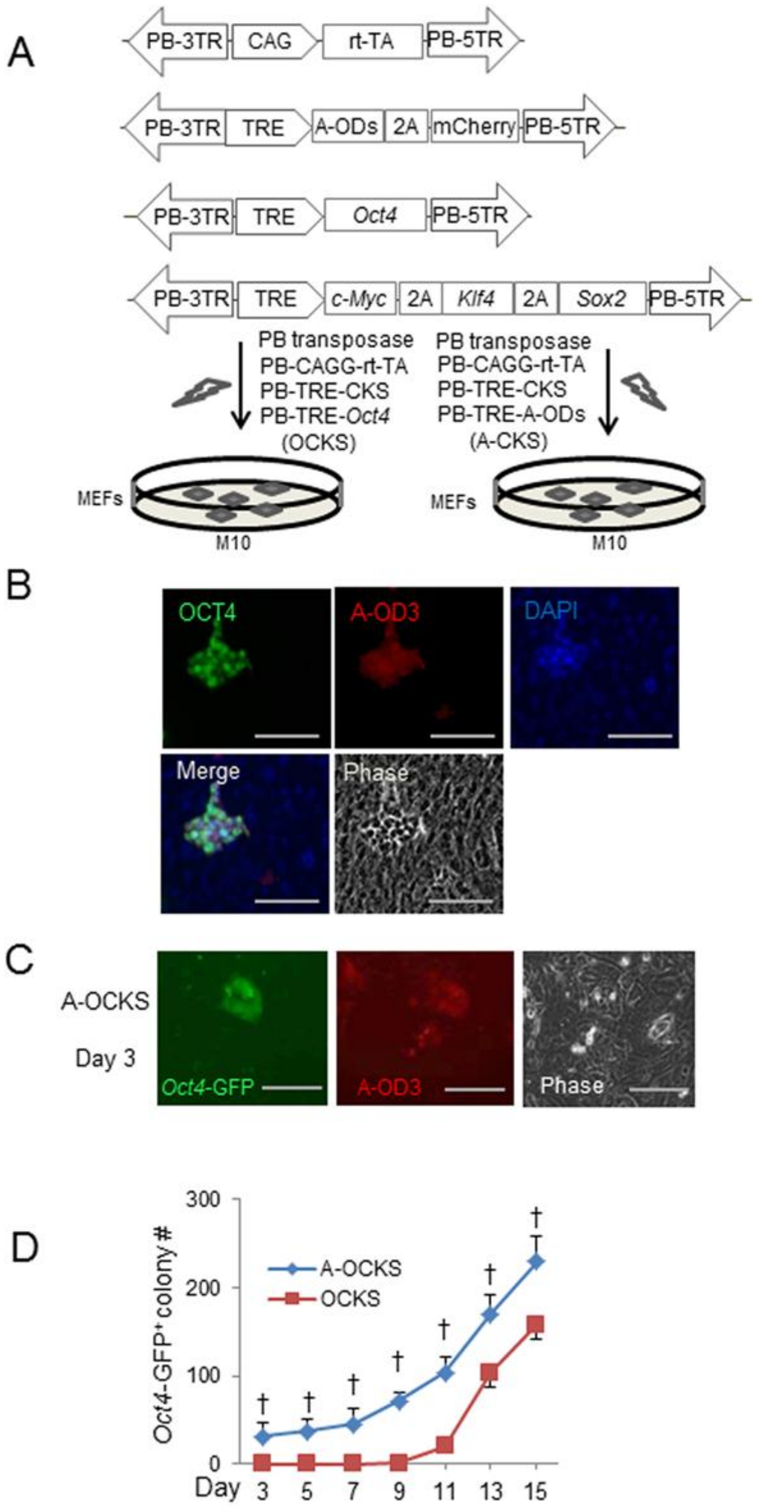
A
PB-3TR
CAG
rt-TA
PB-5TR
PB-3TR
TRE
A-ODs
2A
mCherry
PB-5TR
PB-3TR
TRE
Oct4
PB-5TR
PB-3TR
TRE
c-Myc
2A
Klf4
2A
Sox2
PB-5TR
PB transposase
PB-CAGG-rt-TA
PB-TRE-CKS
PB-TRE-Oct4
(OCKS)
PB transposase
PB-CAGG-rt-TA
PB-TRE-CKS
PB-TRE-A-ODs
(A-CKS)
MEFs
M10
MEFs
M10
B
OCT4
A-OD3
DAPI
Merge
Phase
C
A-OCKS
Day 3
Oct4-GFP
A-OD3
Phase
D
Oct4-GFP⁺colony #
300
200
100
0
A-OCKS
OCKS
Day
3
5
7
9
11
13
15
†

Figure S2. Reprograming MEFs to iPSCs using dTFs. Related to Figure 2. (A) Schematic diagram of using the piggyBac (PB) transposition to deliver exogenous factors into MEFs in reprogramming. Four PB vectors were used: 1. Dox-inducible *c-Myc*, *Klf4* and *Sox2* (CKS). 2. Dox-inducible exogenous *Oct4*. 3. Dox-inducible dTFs. 4. CAG-rtTA. The PB transposase was expressed transiently. Doxcycline induction started immediately after transfection. (B) The immunostaining of endogenous OCT4 protein in MEFs expressing A-CKS for 8 days. (C) Images of a GFP^+ colony from MEFs expressing A-OCKS as early as 3 days after Dox induction. Scale bars: 200.0 μm. (D) GFP^+ colonies reprogrammed from *Oct4*-GFP MEFs by either OCKS or A-OCKS at various time points. Results are representative of three independent experiments and are means ± S.D., n=3. † p<0.05 A-OCKS compared to OCKS.

Figure S3

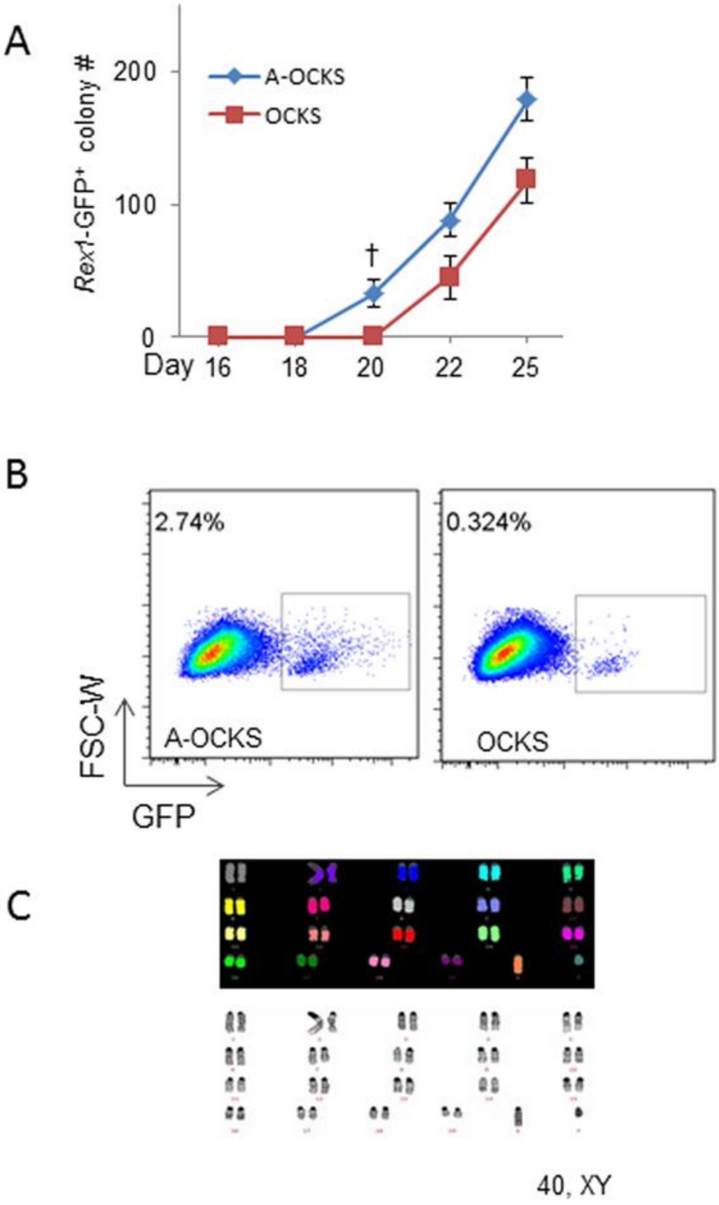

Figure S3. A-OD3 enhances OCKS reprogramming of MEFs to iPSCs. Related to Figure 3. (A) GFP+ colonies reprogrammed from *Rex1*-GFP MEFs by either A-OCKS or OCKS at several time points. (B) Flow cytometric analysis of GFP^{+} cells reprogrammed by A-OCKS and OCKS on day 20. The numbers in the FACS plots are the percentages of GFP+ cells in the total cells. (C) Normal karyotype of iPSCs reprogrammed by A-CKS (passage 16). Results are representative of three independent experiments and are means ± S.D., n=3. † p<0.05 A-OCKS compared to OCKS.

Figure S4

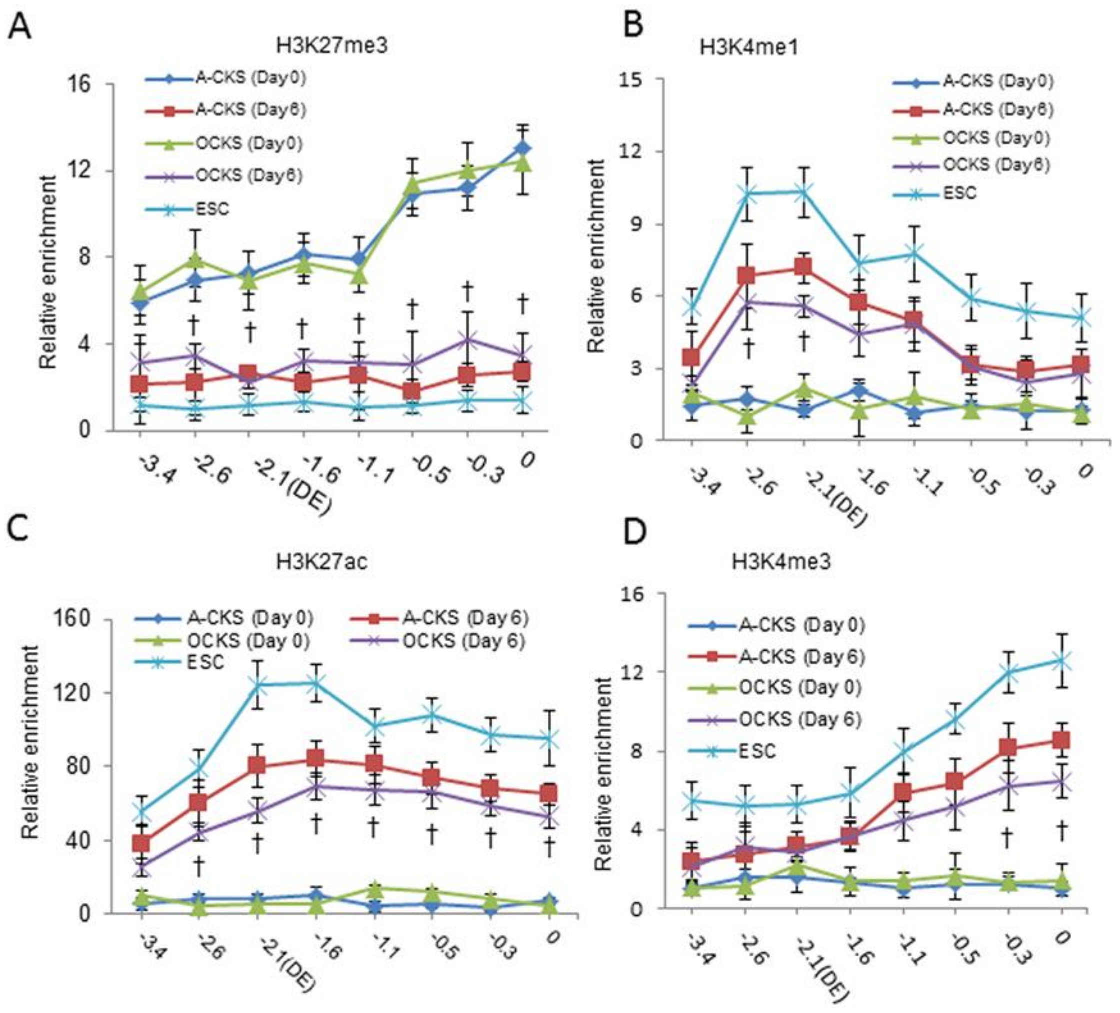

Figure S4. ChIP analysis of histone modifications at the *Oct4* locus in MEFs on day 0 and day 6 of expression of A-CKS (Related to Figure 4): (A) H3K27me3, (B) H3K4me1, (C) H3K27ac and (D) H3K4me3. A genomic region at the *Tyr* locus was used as the unrelated genomic control. The relative enrichments were normalized to the IgG control. Values in x-axis indicate the locations of PCR primers used for ChIP qPCR assay. -0.3: 0.3kb upstream of the TSS. Results are representative of three independent experiments with 3 different lines and are means ± S.D., n=3. † $p<0.05$ Day 6 (OCKS) compared to Day 0 (OCKS).

Figure S5

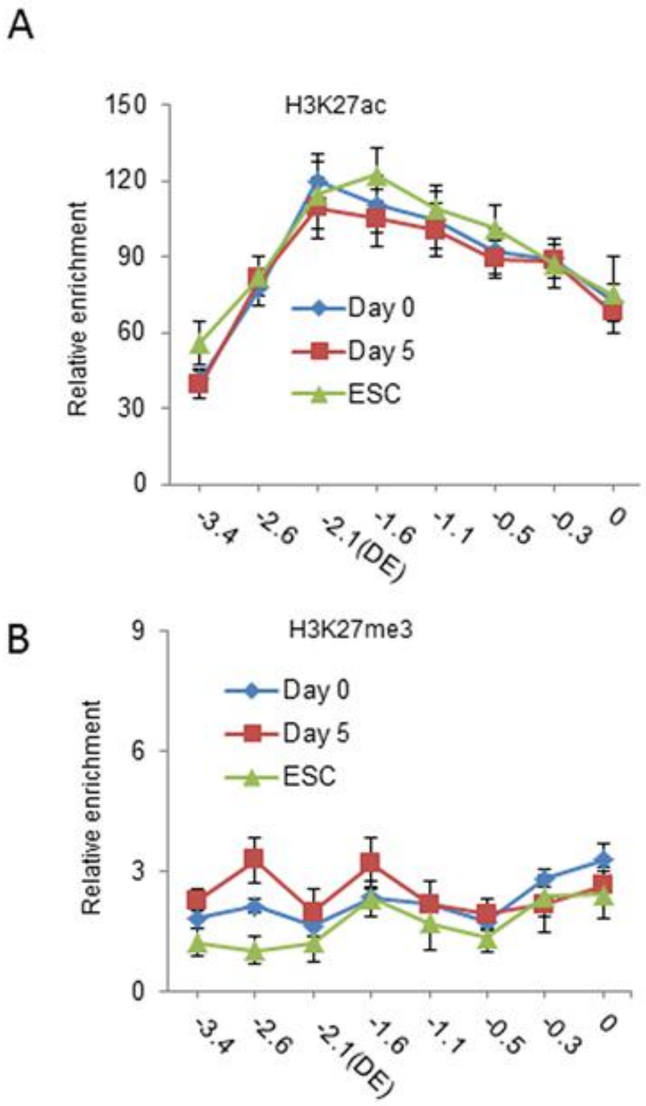

Figure S5. Analysis of epigenetic changes at the *Oct4* locus in ES cells induced by R-OD1 (Related to Figure 5) : (A) H3K27ac and (B) H3K27me3. A genomic region at the *Tyr* locus was used as the unrelated genomic control. The relative enrichments were normalized to the IgG control. Values in x-axis indicate the locations of PCR primers used qCR in ChIP assay. -0.3: 0.3kb upstream of the TSS. Results are representative of three independent experiments with three different lines and are means ± S.D., n=3.

Figure S6

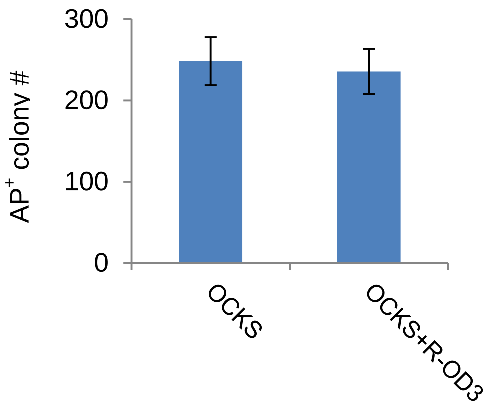

Figure S6. The effect of R-OD3 on reprogramming of MEFs induced by CAG-OCKS. Related to Figure 6. Results are representative of three independent experiments and are means ± S.D., n=3.

Figure S7

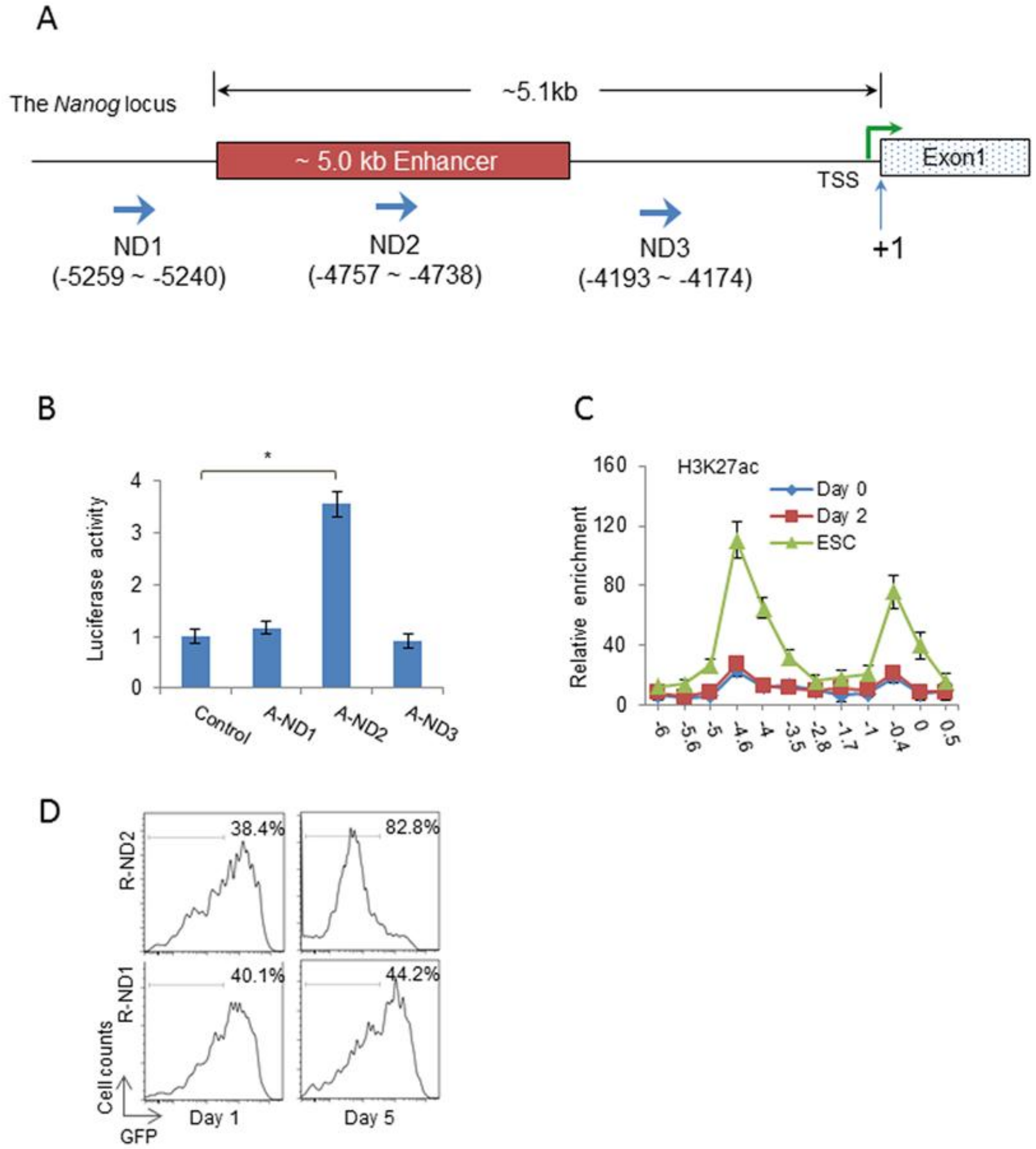
A
The Nanog locus
~5.1kb
~ 5.0 kb Enhancer
Exon1
TSS
ND1
(-5259 ~ -5240)
ND2
(-4757 ~ -4738)
ND3
(-4193 ~ -4174)
+1
B
Luciferase activity
Control
A-ND1
A-ND2
A-ND3
C
H3K27ac
Day 0
Day 2
ESC
Relative enrichment
D
R-ND2
R-ND1
38.4%
82.8%
40.1%
44.2%
Cell counts
GFP
Day 1
Day 5

Figure S7. Regulation of of the *Nanog* locus by dTFs targeting the 5kb enhancer. Related to Figure 7. (A) ND1-3 bind genomic sequences either inside (ND2) or outside (ND1 and ND3) the enhancer. (B) The ~1.0kb DNA fragment of the *Nanog* 5kb enhancer (-5145 to -4154) was cloned into a luciferase reporter. Luciferase activities induced by dTFs in MEFs were measured 48 hours later. (C) H3K27ac levels at the *Nanog* locus were not affected by A-ND1 in EpiSCs. A genomic region at the *Tyr* locus was used as the unrelated genomic control. The relative enrichments were normalized to the IgG control. Values in x-axis indicate the locations of PCR primers used in ChIP qPCR assay. -0.3: 0.3kb upstream of the TSS. (D) Flow cytometric analysis of the repression of the *Nanog* locus by R-ND1 and R-ND2 in Nanog-GFP EpiSCs. Results are representative of three independent experiments with different line and are means ± S.D., n=3. *p<0.01.

Supplementary Tables

Table S1. DNA sequences bound by dTFs. Related to Figure 1 and Figure S1.

dTF name	Target sequence	Location
OD-1	*CGGGACCAGGCTAGGGCAC*	-2371 ~ -2352
OD-2	*ACAGACAGGACTGCTGGGC*	-2146 ~ -2327
OD-3	*AGCCCGACCCTGCCCCTCC*	-2042 ~ -2023
OD-3-25	*AGCCCGACCCTGCCCCTCCCCCCAG*	-2042 ~ -2017
OD-3-37	*AGCCCGACCCTGCCCCTCCCCCCAGGGAGGTTGAGAG*	-2042 ~ -2005
OD-4	*CCCTCTCGTCCTAGCCCTT*	-1927 ~ -1908
OD-5	*TCCACACATGTGCTATGTG*	-1680 ~ -1661
PP1	*CCCCAACCTCCGTCTGGAA*	-140 ~ -121
PP2	*CCCACCCCCACAGCTCTGC*	-89 ~ -70
PP3	*CCTCCACCCACCCAGGGGG*	-37~ -18
PP4	*CAAGGCTAGAGGGTGGGAT*	-36~ -17
ND-1	*CAGAGGCAGACGGGTCTCC*	-5259 ~ -5240
ND-2	*CCCTCCCCAACCCCCATTC*	-4757 ~ -4738
ND-3	*GGCAAAAGTATGTAACTGG*	-4193 ~ -4174

Table S2. AP^+ colonies reprogrammed from *Oct4*-GFP MEFs by dTFs and CKS. Related to Figure 2.

Combination	AP^+ Colonies
CKS	6.6 ± 3.5
CKS+OCT4	463.3 ± 82.4
CKS+AOD1	6.3 ± 2.5
CKS+AOD2	80.6 ± 16.5
CKS+AOD3	261.3 ± 62.5
CKS+AOD4	58.7 ± 12.5
CKS+ AOD5	4.8 ± 2.3
CKS+APP1	282.2 ± 42.4
CKS+APP2	211.3 ± 38.6
CKS+APP3	114 ± 10.8
CKS+APP4	5.5 ± 1.8

Note: One million *Oct4*-GFP MEFs were transfected with Dox-inducible dTFs and CKS. After transfection, MEFs were seeded in M15 plus LIF medium on mitomycin-inactivated STO feeders in 10-cm dishes. Dox (2.0 μg/mL) was added after transfection and was withdrawn on day 14. On day 23, colonies were stained for AP^+ and scored.

Table S3. Primers used for TALE repeat assembly, genomic DNA PCR and RT-PCR. Related to Figure S1, Figure S5, Figure S7, Figure 2, Figure 3, Figure 4, Figure 5 and Figure 7.

Primer	Primer sequence	Purpose
F1	ATATAGATGCCGTCCTAGCGCGTCTCCTGACCCCAGAGCAGGTCGTGG	dTF assembling
F2	*TGCTCTTTATTCGTTGCGTC*GGTCTC*GACTCACCCCAGAGCAGGTCGTG*	dTF assembling
F3	*TGCTCTTTATTCGTTGCGTCGGTCTCGCCTCACCCCAGAGCAGGTCGTG*	dTF assembling
F4	TGCTCTTTATTCGTTGCGTCCGTCTCGATTAACCCCAGAGCAGGTCGTG	dTF assembling
F5	ATATAGATGCCGTCCTAGCGCGTCTCGCTTAACCCCAGAGCAGGTCGTG	dTF assembling
F6	*TGCTCTTTATTCGTTGCGTCGGTCTCGACTCACCCCAGAGCAGGTCGTG*	dTF assembling
F7	*TGCTCTTTATTCGTTGCGTCGGTCTCGCCTCACCCCAGAGCAGGTCGTG*	dTF assembling
F8	*TGCTCTTTATTCGTTGCGTCGGTCTCGATTAACCCCAGAGCAGGTCGTG*	dTF assembling
F9	*ATATAGATGCCGTCCTAGCGGGTCTCGGCTCACCCCAGAGCAGGTCGTG*	dTF assembling
F10	*TGCTCTTTATTCGTTGCGTCGGTCTCGACTCACCCCAGAGCAGGTCGTG*	dTF assembling
F11	*TGCTCTTTATTCGTTGCGTCGGTCTCGCCTCACCCCAGAGCAGGTCGTG*	dTF assembling
F12	*TGCTCTTTATTCGTTGCGTCGGTCTCGATTAACCCCAGAGCAGGTCGTG*	dTF assembling
F13	ATATAGATGCCGTCCTAGCGCGTCTCCGCTGACCCCAGAGCAGGTCGTG	dTF assembling
F14	ATATAGATGCCGTCCTAGCGCGTCTCCTCTGACCCCAGAGCAGGTCGT	dTF assembling
FA	ATATAGATGCCGTCCTAGCG	dTF assembling
R1	TCTTATCGGTGCTTCGTTCTGGTCTCTGAGTCCGTGCGCTTGGCAC	dTF assembling
R2	TCTTATCGGTGCTTCGTTCTGGTCTCTGAGGCCGTGCGCTTGGCAC	dTF assembling
R3	TCTTATCGGTGCTTCGTTCTGGTCTCTTAATCCGTGCGCTTGGCAC	dTF assembling
R4	AAGTATCTTTCCTGTGCCCAGGTCTCTTAAGCCGTGCGCTTGGCAC	dTF assembling
R5	TCTTATCGGTGCTTCGTTCTGGTCTCTGAGTCCGTGCGCTTGGCAC	dTF assembling
R6	TCTTATCGGTGCTTCGTTCTGGTCTCTGAGGCCGTGCGCTTGGCAC	dTF assembling
R7	TCTTATCGGTGCTTCGTTCTGGTCTCTTAATCCGTGCGCTTGGCAC	dTF assembling
R8	*AAGTATCTTTCCTGTGCCCACGTCTCTGAGCCCGTGCGCTTGGCAC*	dTF assembling
R9	*TCTTATCGGTGCTTCGTTCTCGTCTCTGAGTCCGTGCGCTTGGCAC*	dTF assembling
R10	TCTTATCGGTGCTTCGTTCTGGTCTCTGAGGCCGTGCGCTTGGCAC	dTF assembling
R11	TCTTATCGGTGCTTCGTTCTGGTCTCTTAATCCGTGCGCTTGGCAC	dTF assembling
R12	*AAGTATCTTTCCTGTGCCCACGTCTCTCAGCCCGTGCGCTTGGCAC*	dTF assembling
R13	*AAGTATCTTTCCTGTGCCCACGTCTCTCAGACCGTGCGCTTGGCAC*	dTF assembling
R14	*AAGTATCTTTCCTGTGCCCACGTCTCTGAGTCCGTGCGCTTGGCAC*	dTF assembling
RA	AAGTATCTTTCCTGTGCCCA	dTF assembling
OF-3.4	*CATCAGCCCCTCAACTTGCTTTC*	*Oct4* ChIP-qPCR
OR-3.4	CCTTGAGGAAATGCCTCCATGAGA	*Oct4* ChIP-qPCR
OF-2.6	TGACTCTTAAAGGGGGCAGA	*Oct4* ChIP-qPCR
OR-2.6	TGCCTCCTGGGTCTTAGAAA	*Oct4* ChIP-qPCR
OF-2.1(DE)	GGCTGCAGGCATACTTGAAC	*Oct4* ChIP-qPCR
OR-2.1(DE)	GCTACAACCTCCCCACACC	*Oct4* ChIP-qPCR
OF-1.6	TGGTGAAGTCGATGAAGCTG	*Oct4* ChIP-qPCR

OR-1.6	GAGCTGTTGGCTAGGGTCAG	*Oct4* ChIP-qPCR
OF-1.1	TTAGTGTCTTTCCGCCAGCACAG	*Oct4* ChIP-qPCR
OR-1.1	CCTCAGATGGAGATACCCTGCT	*Oct4* ChIP-qPCR
OF-0.5	GCACTTCTCTGGGGTCTCTG	*Oct4* ChIP-qPCR
OR-0.5	ACCCACCCGTCTAGAGTCCT	*Oct4* ChIP-qPCR
OF-0.3	AATTGGCACACGAACATTCA	*Oct4* ChIP-qPCR
OR-0.3	GTCCTTACAGCCCACTCAGC	*Oct4* ChIP-qPCR
OF-0	CCTAAGGGTTGTCCTGTCCA	*Oct4* ChIP-qPCR
OR-0	AGCGCTATCTGCCTGTGTCT	*Oct4* ChIP-qPCR
NF-6	CAGATTCATGGTCCCTACCACT	*Nanog* ChIP-qPCR
NR-6	GTTCTTTTGCCAGCATCATCAG	*Nanog* ChIP-qPCR
NF-5.6	TACTGCCTGGCTGTGTGTGGGTGC	*Nanog* ChIP-qPCR
NR-5.6	AGCTCAGGCCCACAAAGCAGTTGGAGC	*Nanog* ChIP-qPCR
NF-5	ACCTGTCCCTAGTCCCCGCTCCTTT	*Nanog* ChIP-qPCR
NR-5	TGGCTGGTAGCCAAAAGGCAGGCT	*Nanog* ChIP-qPCR
NF-4.6	TGACTCCGTGGACCCAGAGGCAAGT	*Nanog* ChIP-qPCR
NR-4.6	AACCCTAGGTGTGTCCCAAGGGCGA	*Nanog* ChIP-qPCR
NF-4	TGTTAGCGATGGGCCCCGTGCTTT	*Nanog* ChIP-qPCR
NR-4	ACCCGCTGAGCATTCTGCCACTCACA	*Nanog* ChIP-qPCR
NF-3.5	CCATTCTTCGTTTAAGCAAACC	*Nanog* ChIP-qPCR
NR-3.5	AGAATGAATAGGCCGTTCAAAA	*Nanog* ChIP-qPCR
NF-2.8	TGAAAATGGAATCACTGGAACC	*Nanog* ChIP-qPCR
NR-2.8	CCCATCTCTTAAGCCCTCCTCT	*Nanog* ChIP-qPCR
NF-1.7	GAGGCATTGCAGGCTTTAGTG	*Nanog* ChIP-qPCR
NR-1.7	TTACCACCACTTCCCTTCTTCC	*Nanog* ChIP-qPCR
NF-1	AGGACGGCCCTTCCCTCTCTGCT	*Nanog* ChIP-qPCR
NR-1	ACGTACCCGAGACTGGCCTCACAGT	*Nanog* ChIP-qPCR
NF-0.4	ACGCTGAGTGCTGAAAGGAAAGCCGTG	*Nanog* ChIP-qPCR
NR-0.4	TGCACCTCCAGACCCTGGCGATCT	*Nanog* ChIP-qPCR
NF0	TCTGTGGGAAGGCTGCGGCTCACT	*Nanog* ChIP-qPCR
NR0	TGTGGGGACCAGGAAGACCCACACT	*Nanog* ChIP-qPCR
NF0.5	AGATCCGGGACACAGGACGGAGCA	*Nanog* ChIP-qPCR
NR0.5	AGACACCACTCACTGCCCCCGAACA	*Nanog* ChIP-qPCR
*Tyr*F	GCTTCTTCATCCTGCTGGTC	*Nanog* ChIP-qPCR
*Tyr*R	GGGAGCCATTCTCATTCAAA	*Nanog* ChIP-qPCR
Oct4-DMR-F	TGGGTTGAAATATTGGGTTTATTT	Bisulfite Sequencing
Oct4-DMR-R	CTAAAACCAAATATCCAACCATA	Bisulfite Sequencing
Nanog-DMR-F	GATTTTGTAGGTGGGATTAATTGTGAATTT	Bisulfite Sequencing
Nanog-DMR-R	ACCAAAAAAACCCACACTCATATCAATATA	Bisulfite Sequencing
Gapdh-F	CTGCACCACCAACTGCTTAGC	RT-PCR
Gapdh-R	GGAAGGCCATGCCAGTGA	RT-PCR
A-DE25-F	TTGGGATATTCACAGCAGCAGCAG	RT-PCR
A-DE25-R	ACACCGACGATCGCCTCATGTGTG	RT-PCR
PB-CKS-F	GCAGACGAGCACAAGCTCACCTC	RT-PCR
PB-CKS-R	GGAAGACGAGGATGAAGCTGAC	RT-PCR

Table S4. Applied Bioscience predesigned and custom-designed TaqMan probes for real-time RT-PCR of mouse genes. Related to Figure 2, Figure 3, Figure 5, Figure 6, Figure 7 and Figure S1.

Predesigned qPCR assays	Target	Applied Bioscience gene name
Mm02384862_g1	*Nanog*	Nanog homeobox
Mm03053975_g1	*Rex1*	Zinc finger protein 42
Mm00836373_g1	*Stella*	Developmental pluripotency associated 3
Mm99999915_g1	*Gapdh*	Mouse GADPH endogenous control
Mm00508531_m1	*Tcf19*	Transcription factor 19
Mm00461542_m1	*Cchcr1*	Coiled-coil alpha-helical rod protein 1
Mm01275264_g1	*H2Q-10*	Histocompatibility 2, Q region locus 10
Mm01702237_m1	*Kcnk18*	Potassium channel, subfamily K, member 18

Table S5. Prime pairs used in monomer PCR for dTF assembling. Related to Figure S1.

F1/R1 M1	F2/R2 M2	F3/R3 M3	F4/R4 M4	F5/R5 M5	F6/R6 M6	F7/R7 M7	F8/R8 M8	F9/R9 M9	F10/R10 M10	F11/R11 M11	F12/R12 M12
F13/R1 M13	F2/R2 M14	F3/R3 M15	F4/R4 M16	F5/R5 M17	F6/R6 M18	F7/R7 M19	F8/R8 M20	F9/R9 M21	F10/R10 M22	F11/R11 M23	F12/R13(R14,) M24
F14/R1 M25	F2/R2 M26	F3/R3 M27	F4/R4 M28	F5/R5 M29	F6/R6 M30	F7/R7 M31	F8/R8 M32	F9/R9 M33	F10/R10 M34	F11/R11 M35	F12/R14 M36

Note: M1: monomer #1. For M24 PCR, F12/R14 and F12/R13 primer pairs were used for 24 and 36 repeats assembling, respectively.

Supplementary experimental procedures

dTF assembling. High-fidelity Herculase II polymerase (Stratagene) was used for PCR-amplifying the repeat monomers (Table S1). Monomers were amplified in 50µl PCR reactions according to manufacturer's protocol. After completion of the PCR reaction, monomers were purified and eluted in 50µl of ddH_2O. The PCR products were then digested using BsaI or BsmBI for 2 hours. Tetramers were first constructed by ligating four individual digested monomers together using 300units of T7 ligase (Enzymatics). The ligation was incubated at room temperature for 1.5 hour. The ligation reactions were run on a 2% agarose gel and products of the correct size (450 bp) were cut out and amplified by PCR again. The amplified tetrameric repeats were purified and ligated into BluntII-TOPO cloning vector according to manufacturer's protocol. After sequencing confirmation, three tetramers were digested using BsaI or BsmBI for 2 hours and the expected fragments were purified, and ligated to form 12-mers. TALEs were then generated by ligating two or three 12-mer repeats with the backbone vector.

Plasmid Vector Construction. To make PB-TRE-CKS and PB-CAG-OCKS vectors, the TRE promoter was amplified from pTight (Clontech) and the CAGG promoter was amplified from a pBluescript-CAG vector, and cloned into a PB-bpA vector. cDNAs of the mouse *Oct4, c-Myc, Klf4* and *Sox2* were tandemly cloned into the PB-TRE and PB-CAG transposon vectors to generate PB-TRE-CKS and PB-CAG-OCKS accordingly

Preparation of MEF Cells for Reprogramming. MEFs were prepared from 13.5-day postcoitum mouse embryos. To minimize variation among embryos, MEFs from several embryos with the same genotype were mixed together for expansion in M10 media. MEFs were passaged once before they were counted, divided into aliquots, and cryopreserved. Approximately 1×10^6 frozen MEFs were thawed and plated onto one gelatinized 15-cm tissue culture plate. MEFs were trypsinized and collected for electroporation at 80% confluence. M10: knockout DMEM, 10% FBS (HyClone), 1×glutamine penicillin-streptomycin (Invitrogen), and 1× NEAA (Invitrogen).

Transfection of MEFs and reprogramming to iPSCs. MEF transfection was performed using an Amaxa Nucleofector machine (Lonza) according to the manufacturer's protocol (program A-023). One million MEFs and 5.0 **µ**g DNA (1.0 µg PB-transposase and 4.0 µg PB

transposons) were used in each electroporation reaction. MEFs were seeded in M15 plus LIF on mitomycin-inactivated STO feeders in 10-cm dishes. Doxcycline (2.0 **μ**g/mL) was added after transfection for transgene induction and withdrawn on day 14. Transfection efficiency was measured by electroporating a PB-CAG-mCherry plasmid to MEFs, and counted mCherry$^+$ cells vs. total live cells two days of transfection. About ~40% MEFs survived electroporation, and around 5% of the survived MEFs expressed mCherry counted two days after transfection. Similarly, the PB transposition efficiency was calculated by counting mCherry$^+$ MEFs vs. total live cells 10 days after transfection, and was independently estimated by using the PB transposon carrying a Puro^r cassette and scoring Puro^r fibroblast cell colonies. iPSC colonies were picked on day 23-25 and expanded in standard mouse ES cell culture conditions. To analyze GFP$^+$ cells from *Oct4*-GFP MEFs for their reprogramming potential, GFP$^+$ cells were harvested by flow sorting, and 600 of them were plated to a well of 6-well dish on STO feeders. Dox was taken off from the culture medium 3 days after re-plating accounting for total induction time of 14 days, identical to the regular reprogramming experiments described in Figure 2A, and the AP$^+$ (alkaline phosphatase) colonies were scored on day 23 post transfection.

EpiSCs culture. EpiSCs were routinely cultured in N2B27/Activin/bFGF: DMEM/F-12 (Gibco, 21331-020) medium supplemented with N2, B27, human Activin A (20 ng/ml; Peprotech) and bFGF (12 ng/ml; Invitrogen). For N2B27/2i/LIF, DMEM/F-12 (Gibco, 21331-020) medium were supplemented with N2, B27, LIF (1000 U/ml), the Mek inhibitor PD0325901 (1 μM; Stemgent) and the Gsk3beta inhibitor CHIRON99021 (3 μM; Stemgent). In the experiment that the TALE activators were transfected to EpiSCs for *Nanog* expression, *Nanog* mRNA levels were quantitated by qRT-PCR 48 hours after transfection and Dox induction. As the positive control, EpiSCs were also transfected with a PB vector expressing the *Nanog* cDNA. For reprogramming EpiSCs, two days after A-ND2 expression, the culture medium was changed from N2B27/FGF/Activin to N2B27/2i/LIF (or 2i/LIF) for 12 days, which preferentially selects and maintains naïve pluripotent stem cells

ChIP Analysis. For 10 million ES cells cultured in a 10-cm dish and 5 million differentiated ES cells cultured in a 15-cm dish: cells were collected 2 days after transfection of TALE-expressing plasmids and trypsinized for 5-8 min, trypsin was quenched by addition of 10 ml media containing 10% FBS. Cell suspension was diluted to 40 ml with PBS and cells were fixed for 12 min by formaldehyde at a final concentration of 1%. Cells crosslinking was quenched by 2.5 M glycine (0.125 M final concentration) before cells were incubated on ice.

Crosslinked cells were spun at 600 × g for 5 min, nuclei were prepared by consecutive washes with P1 buffer (10 mM Tris pH 8.0, 10 mM EDTA [pH 8.0], 0.5 mM EGTA, 0.25% Triton X-100) followed by P2 buffer (10 mM Tris pH 8.0, 1 mM, EDTA, 0.5 mM EGTA, 200 mM NaCl). Pellets were resuspended in 2 ml of ChIP lysis buffer (50 mM HEPES/KOH, pH=7.5, 300 mM NaCl, 1 mM EDTA, 1% Triton X-100, 0.1% DOC, 0.1% SDS, protease inhibitors complete mini (Roche)) and then sonicated using BioRuptor (Diagenode) and pulsed with 15 cycles of 30 seconds sonication and 30 seconds rest. DNA was sheared to the size range between 500 and 1000 bp (confirmed on agarose gel). IgG (Cell Signalling, 2729S) and antibodies for the HA tag (Cell Signalling, 71-5500), H3K4me3 (Cell Signalling, C42D8), H3K4me1 (Diagenode, Cs-037-100), H3K27ac (Abcam, ab4729) and H3K27me3 (Cell Signalling, C36B11) were used in ChIP analysis. Primers for qRT-PCR are listed in Table S3.

Luciferase Assay Luciferase reporter plasmids (5.0 **μ**g), TK-Renilla (0.5 **μ**g) (Promega) were transfected into MEFs, together with expression vectors of reprogramming factors (5.0 **μ**g). Forty-eight hours after transfection, cells were lysed with passive lysis buffer (Promega). Luciferase activities were measured with a Dual- Luciferase reporter assay system (Promega) according to the manufacturer's protocol.

Alkaline phosphatase staining. Cells were fixed in citrate–acetone–formaldehyde and stained using the Alkaline Phosphatase kit (Sigma-Aldrich) according to the manufacturer's instructions.

Flow cytometry. Flow cytometry was performed using a BD Fortessa analyser with subsequent data analysis using FlowJo 7.6.5 software. Cell sorting was performed using a MoFlo XDP (BD) cell sorter. mCherry and GFP were excited using 561 nm and 488nm laser and detected using a 610/20 and 530/30 filter.

Immunostanning. For dual staining of SSEA-1 and NANOG, mouse iPSCs were fixed in 4% PFA/PBS solution, blocked in PBS solution with 3% goat serum and 1% BSA, incubated with anti–SSEA-1 antibody (Abcam) at 4 °C overnight. Cells were then rinsed with PBS solution, incubated with Alexa 488-conjugated goat anti-mouse IgM (Invitrogen) for 1 h at room temperature. After permeabilization with PBST (PBS solution with 0.3% Triton), cells were incubated with anti-NANOG antibody (Abcam) at 4 °C for overnight. In the third day, cells were rinsed with PBST, incubated with Alexa 594-conjugated goat anti-rabbit IgG (Invitrogen) for 1 h at room temperature, and counterstained with DAPI.

RT-PCR. RNA was isolated using the RNeasy Mini Kit (Qiagen). The samples were

subsequently quantified and treated with gDNA Wipe-Out buffer (Qiagen). First-strand cDNA was prepared by using the QuanTect Kit (Qiagen). For each RT-PCR, we used 50 to 100 ng of cDNA. Standard PCR conditions were: 94 °C for 30 s, 60 °C for 30 s, and 68 °C for 30 s for 30 cycles. For endogenous *Oct4* gene expression, custom designed TaqMan Gene Expression probe sets were used: Forward, CTCTCCCATGCATTCAAACTGA; reverse, CCCTTGCCTTGGCTCACA; Probe, CACCAGCCCTCCCT. The information of other probe sets and primers were given in Table S3 and Table S4. All quantitative PCR was performed in a 9700HT Fast Real-Time PCR System (Applied Biosciences). Gene expression was determined relative to mouse *Gadph* using the **ΔΔ**Ct relative quantification method.

Bisulfite Genomic Sequencing. Bisulfite treatment was performed by using the EpiTect Bisulfite Kit (Qiagen) according to the manufacturer's recommendations. PCR primers are listed in Table S3. Amplified products were cloned into pGEM-T-easy vectors (Promega). Randomly selected clones were sequenced with the M13 forward and M13 reverse primers for each promoters.

***In Vitro* Differentiation of iPSCs.** Mouse iPSCs were harvested by trypsinization and transferred to six wells at 1 to 1.5×10^4 cells/cm^2 in N2B27 medium. Medium was changed every other day. On day 6, cells were fixed and immunostained for **α**-smooth muscle actin (SMA), ß-tubulin and **α**-fetoprotein (AFP).

Statistical Analysis. Statistical significance was determined using a Student's t test with two tailed distribution. p-values less than 0.05 were considered as significant. Data are shown as mean and SD.
